# AlphaFold reveals how pathogenic *Leptospira* use cross-kingdom thiol-disulfide exchange to evade the complement membrane attack complex

**DOI:** 10.1128/mbio.00878-26

**Published:** 2026-06-15

**Authors:** Abbie Hinds, Emily Johnson, Gareth Staton, Ferdinand N. Njume, Hayley E. Crosby-Durrani, Jarlath E. Nally, Stuart Carter, Daniel J. Rigden, Nicholas J. Evans

**Affiliations:** 1 Institute of Infection, Veterinary and Ecological Sciences, University of Liverpool, Leahurst Campus, Chester 105724, Neston, United Kingdom; 2 Institute of Systems, Molecular and Integrative Biology, University of Liverpool 4591, Liverpool, United Kingdom; 3 Computational Biology Facility, LIV-SRF, MerseyBio, University of Liverpool 4591, Liverpool, United Kingdom; 4 Infectious Bacterial Disease Research Unit, National Animal Disease Centre, United States Department of Agriculture, Agricultural Research Service 57837 , Ames, Iowa, USA; Washington University in St. Louis, St. Louis, Missouri, USA

**Keywords:** *Leptospira*, AlphaFold2, complement evasion, membrane attack complex (MAC), thiol-disulfide exchange, cross-kingdom, host-pathogen interactions

## Abstract

Leptospirosis is a globally significant severe infectious disease that affects a range of mammals. To understand its complex aetiopathogenesis, *Leptospira* spp. are classified as pathogenic or saprophytic, with serum resistance a key differentiator of virulence. LIC13259 is a leptospiral surface lipoprotein that binds C8 of the host complement system; however, the molecular basis of this interaction has remained unclear. AlphaFold2 structural predictions, together with protein engineering, biochemical, and immunological validations, revealed that LIC13259 binds specifically to the C8γ subunit via thiol-disulfide exchange. Binding assays confirmed that LIC13259 from pathogenic, but not saprophytic, *Leptospira* adheres to C8γ, preventing C8α binding, and enabling membrane attack complex evasion. Substitution of cysteine-133 with an alanine abolished both C8γ binding and complement inhibition. Removal (alanine substitution) of the target cysteine from C8γ prevented pathogenic LIC13259 binding, whereas removing LIC13259 cysteine-108, which forms a predicted intramolecular disulfide bond with cysteine-133, enhanced C8γ binding. These results identify an unprecedented cross-kingdom intermolecular disulfide bond mediating complement evasion and highlight the value of AlphaFold structural modeling, combined with targeted mutagenesis and biochemical validation, for discovering host-pathogen protein-protein interactions. The discovery of this novel immune evasion mechanism may allow recognition of this unique host-pathogen interaction in a range of diseases, while therapeutics and vaccines targeting such cross-kingdom disulfide-mediated mimicry may enable future infectious disease control.

**IMPORTANCE:**

Leptospirosis is a globally important zoonotic disease of humans and animals, caused by the bacteria Leptospira. A central determinant of virulence is the ability for these bacteria to evade complement, a host defense system that assembles the membrane attack complex to eliminate pathogens. Here, we elucidate a novel immune evasion mechanism in which the leptospiral surface protein LIC13259 forms a disulfide bond with complement component C8γ, preventing binding of C8α. This interaction disrupts the membrane attack complex assembly and promotes bacterial survival. To our knowledge, this represents the first example of a cross-kingdom disulfide bond mediating bacterial pathogenesis. These findings provide novel insights into leptospiral immune modulation and demonstrate the power of AlphaFold-based structural predictions to reveal unique host-pathogen interactions.

## INTRODUCTION

Leptospirosis is a globally significant zoonotic disease affecting a wide range of mammals, including cattle and humans (1). The disease is caused by *Leptospira* bacteria—a genus encompassing two clades, pathogenic species (P) and saprophytic species (S), that are free-living in the environment and not disease-associated (2, 3). Globally, ~1 million cases of human leptospirosis occur per year, leading to 60,000 deaths (4). Both cattle and humans can suffer from acute disease, which causes flu-like symptoms and can lead to severe kidney damage and death (5). In cattle, chronic disease is associated with reproductive loss and milk drop syndrome, as well as zoonotic risk. The estimated cost of poor reproductive performance is €231 per cow per year (6), with the economic burden considered substantially greater in tropical countries where there is a higher disease presence. Moreover, increased disease burden in tropical regions has been associated with the greater diversity of pathogenic serovars, which makes disease control more difficult (5, 7).

Cattle serve as reservoir hosts that harbor *Leptospira* in the kidneys and are key to the enzootic disease cycles. These bacteria can be shed in urine leading to soil and water contamination. Leptospires can infect hosts via mucous membranes or abrasions/cuts upon contact with contaminated animals or environments (1, 7, 8). Human infections usually result from occupational contact with infected livestock, recreational exposure to natural water sources, or increased environmental contamination following rainy seasons (8). Due to recent changes in climate and increased flooding frequency, there is an increased risk of transmission for both cattle and humans (8, 9).

*Leptospira* are currently subdivided into 74 genomic species, which can be further subdivided antigenically into >250 serovars and 30 serogroups (10, 11). Infections are typically caused by eight pathogenic species within subclade P1, with the most prevalent species being *Leptospira interrogans* in humans and *Leptospira borgpetersenii* in cattle (1, 2, 11). Infection of cattle with *L. interrogans* has also been reported, albeit at a lower global prevalence than *L. borgpetersenii* (12). Subclade P2 contains species that potentially cause disease, previously referred to as intermediate species (11). Of the saprophytic species, *Leptospira biflexa* (subclade S1) is the most studied. Genetic comparisons have identified the absence of certain genes in *L. biflexa* compared to *L. borgpetersenii* and *L. interrogans*; consistent with their demonstrated roles in pathogenesis (13). However, it is poorly understood how different *Leptospira* species cause disease and interact with the host immune system (13), with serovars indicating host specificity (14, 15). A central theme to understanding leptospiral pathogenesis has included a focus on serum resistance, with the ability to survive complement-mediated killing considered a key differentiator between pathogenic and saprophytic leptospires (16).

The innate immune system acts as the host’s first line of defense against invading leptospires, with complement system activation occurring in the first few hours following infection (16–18). The complement system is activated via three pathways: the classical, alternative, and lectin pathways, ultimately leading to cleavage of C3 (19, 20). C3b forms part of C5 convertase, resulting in the formation of the membrane attack complex (MAC) (21). C5b binds to the pathogen surface and initiates the assembly of the MAC by recruiting C6 and then C7. Following C7 association, the complex undergoes a conformational change, allowing insertion into the pathogen membrane (19, 20). C8 is composed of a disulfide-linked C8α-γ heterodimer and a non-covalently associated C8β subunit. The C8β subunit binds to the C5b-8α-γ complex, allowing C8α-γ to insert into the lipid membrane via C8α binding to C8β. Polymerization of 10-16 C9 molecules is induced by the binding of C9 to C8α, resulting in the pore-forming structure of the MAC, which subsequently causes cell lysis (19–22). Inhibition of MAC formation via leptospiral protein binding to complement terminal components contributes to pathogenic *Leptospira* host immune evasion. LIC13259 is a *Leptospira* lipoprotein associated with pathogenesis that binds C7, C8, and C9 (23), with more LIC13259 binding observed to C8. Although it has been indicated that LIC13259 inhibits MAC formation, the molecular binding mechanism behind the inhibition remains unknown.

Bacterial host-pathogen interactions are an evolutionary arms race, substantially driven by protein-protein interactions (PPIs). These interactions are central to infection biology, enabling pathogens to hijack host cellular processes, evade immune responses, and promote survival and proliferation. With constant selection at the host-pathogen interface, new approaches are needed to discover antagonistic interactions and develop therapeutics (24). Traditional experimental approaches to uncover PPIs are labor-intensive and often limited in throughput or applicability to cross-species interactions (25). Recent advances in artificial intelligence, notably AlphaFold2 (AF2) and its recent successor, AlphaFold3 (AF3), have emerged as methods that can predict protein structures, trained on multiple sequence alignments (MSAs) and structural information. These tools not only accurately predict individual protein structures but have also been shown to be highly effective in predicting PPIs (26), even cross-kingdom interactions that lack the intermolecular sequence covariance resulting from evolutionary pressure to maintain an interaction (27, 28). This opens new avenues for *in silico* screening of potential host-pathogen PPIs and functional characterization. Moreover, the advent of gene synthesis expedites targeted mutagenesis, which, combined with structural predictions and biochemical validations, enables the dissection of molecular interactions.

In this study, we apply AF2 to characterize the structure and mechanism of LIC13259 in the context of MAC binding, with the aim of uncovering the molecular strategies employed by pathogenic *Leptospira* to evade complement-mediated killing. We provide insights into a novel mechanism, showing that pathogenic *Leptospira* LIC13259 forms a covalent intermolecular disulfide bond with C8γ via thiol-disulfide exchange, disrupting C8α-γ association and inhibiting MAC assembly, with broader implications for understanding bacterial pathogenesis.

## RESULTS AND DISCUSSION

### LIC13259 sequences segregate according to pathogenicity

**Fig 1:**
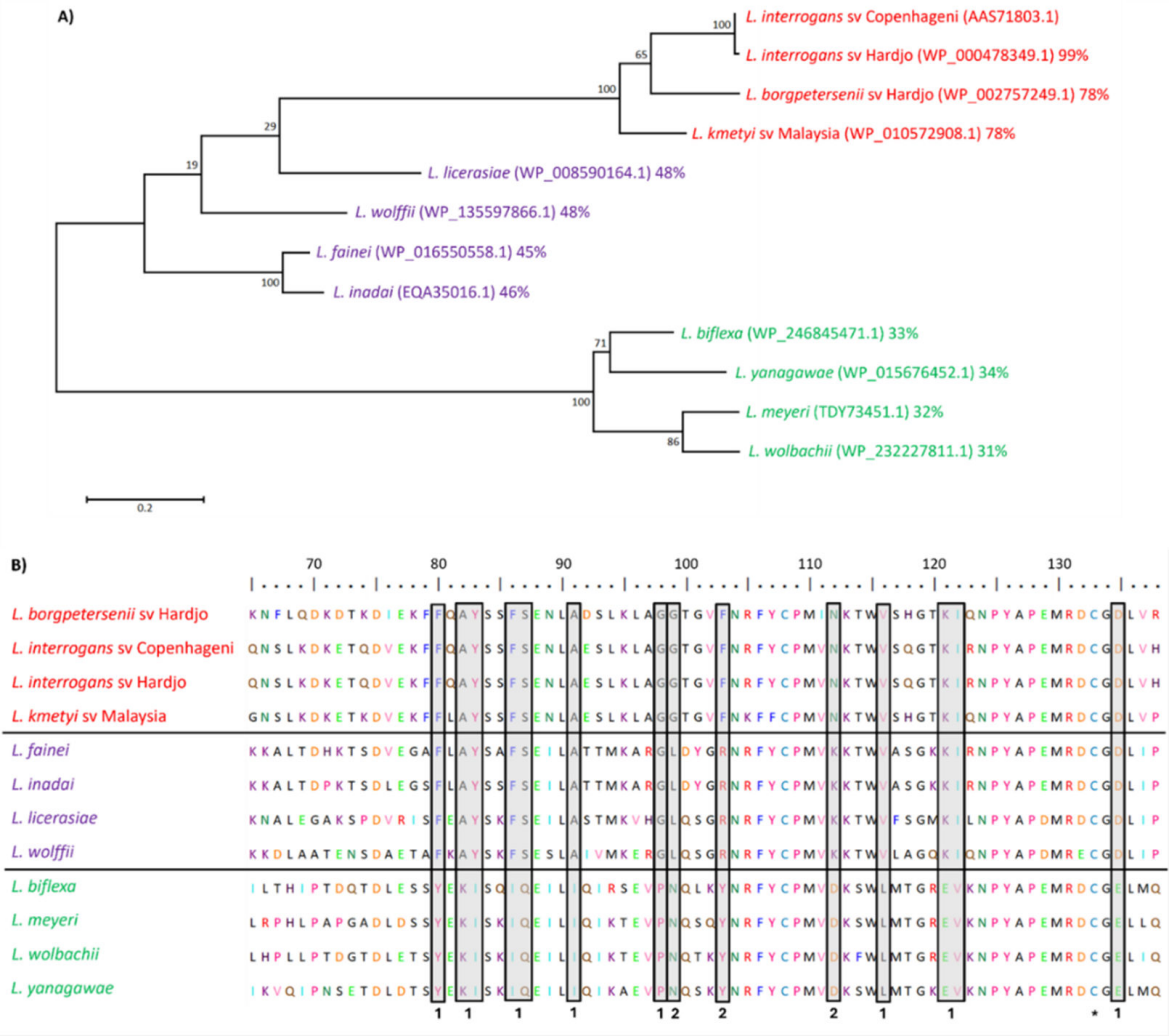
LIC13259 sequence analyses resolve divergence according to leptospire pathogenicity subclades. LIC13259 sequences from pathogenic species subclade P1 (red) *L. interrogans* sv Copenhageni, *L. interrogans* sv Hardjo, *L. borgpetersenii* sv Hardjo, and *L. kmetyi* sv Malaysia; pathogenic species subclade P2 (purple) *L. inadai*, *L. fainei*, *L. licerasiae*, and *L. wolffii*; and saprophytic species subclade S1 (green) *L. biflexa*, *L. yanagawae*, *L. meyeri*, and *L. wolbachii* were taken from the non-redundant protein sequence database at the NCBI; accession numbers in brackets; percentage conservation relative to *L. interrogans* sv Copenhageni. The phylogenetic tree (**A**) was produced in MEGA6. The scale represents the branch length to indicate genetic change (number of amino acid substitutions per site). Annotations above branches display the bootstrapped value. Sequences were aligned and annotated (**B**) using BioEdit 7.7. Sites of interest are highlighted and labeled 1 (amino acid conservation between P1 and P2 subclades) or 2 (amino acid differences between P1, P2, and S1 subclades). *Conserved cysteine between strains at 133.

The pathogenicity of leptospires varies substantially, with strains separated into two groups (pathogenic or saprophytic) categorized by disease-causing ability (2, 3). LIC13259 sequences from four *Leptospira* species of each pathogenicity group were compared (Fig. 1). Phylogenetic analysis of LIC13259 orthologs (Fig. 1A) revealed three groups, separated in accordance with pathogenicity. Sequence alignment of LIC13259 across species identified amino acid substitutions that distinguish pathogenicity groups (Fig. 1B). At some positions, labeled 1, both P1 and P2 subclades translate the same amino acids, suggesting functional conservation between these two groups. In contrast, S1 displays substitutions at these sites, suggesting differences in LIC13259 sequences may influence protein structure and function, affecting host interactions or virulence mechanisms and pathogenicity. At three further sites, the P1, P2, and S1 species display clear amino acid differences between pathogenicity groups (labeled 2).

*Leptospira* proteins are known to evade the immune response through interactions with the MAC. Previous research found LIC13259 binds to C8 (22), and although it has been indicated LIC13259 inhibits MAC formation, the specific molecular mechanism remains unknown. Moreover, research indicates that the same serovars interact differently between hosts (14). Therefore, we utilized AF2 to uncover the mechanism by which LIC13259 interacts with human and bovine C8 and subsequently experimentally validated these predictions. Understanding how leptospires interact with the MAC will provide insight into *Leptospira* pathogenicity and lead to the development of more targeted therapeutics.

### LIC13259 preferentially binds to C8γ

To evaluate differences between pathogenicity groups and take into consideration differing host immune responses, analysis was conducted with the LIC13259 orthologous sequence from P1 strains *L. borgpetersenii* and *L. interrogans*, P2 strains *L. kmetyi* and *L. wolffii*, and S1 strain *L. biflexa*, and their ability to bind host C8. Previous research has shown that LIC13259 binds to complement component C8 to impair the host immune response. However, C8 is a multimer, consisting of α, β, and γ subunits, and it is unknown which subunit LIC13259 targets. We first sought to identify which subunit of C8 LIC13259 might bind to by screening against individual subunits (in addition to full C8, see Fig. S1 and Table S1).

**Fig 2:**
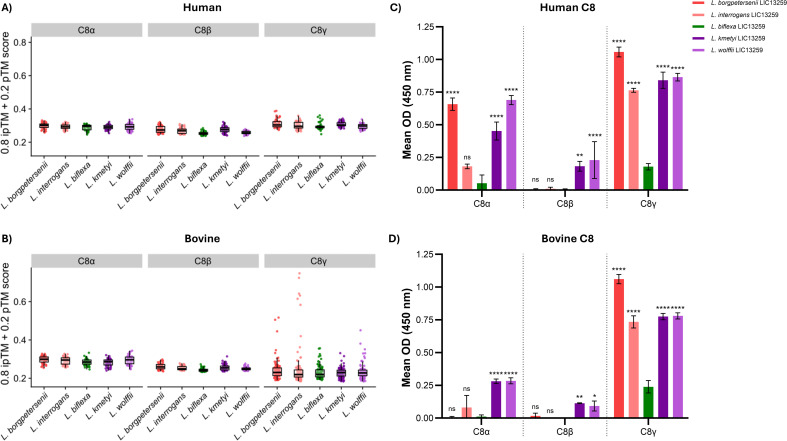
LIC13259 preferentially binds to the C8γ subunit. Box plots showing the distribution of AlphaFold2 0.8 ipTM + 0.2 pTM confidence scores for the predicted multimers between *Leptospira* LIC13259 orthologs and human (**A**) or bovine (**B**) C8 subunits. Each individual point represents a model (*n* = 50). Points are colored by species, with pathogenic P1 species shown in red, pathogenic P2 species shown in purple, and saprophytic S1 species shown in green. The center line is the median value, the box limits are the upper and lower quartiles, and the whiskers are 1.5 × the interquartile range. Binding of LIC13259 from *Leptospira* pathogenic P1 species *L. borgpetersenii* and *L. interrogans*, saprophytic S1 species *L. biflexa*, and pathogenic P2 species *L. kmetyi* and *L. wolffii* to human (**C**) and bovine (**D**) C8 subunits (α, β, or γ) was qualitatively assessed by ELISA. Purified recombinant C8 subunits (5 µg/mL) were coated onto 96-well plates, and binding of recombinant LIC13259 proteins (12 µM) was measured by determining the optical density (OD) at 450 nm. Data are presented as *N* = 2 ± SD. Data were statistically analyzed using a two-way ANOVA followed by Tukey’s multiple comparisons *post hoc* test (****P < 0.0001; **P < 0.01; *P < 0.05; ns = not significant compared to *L. biflexa* binding to the corresponding C8 subunit).

Although LIC13259 oligomerization *in vivo* is possible, we observed LIC13259 as monomeric on native-PAGE and SDS-PAGE. Therefore, LIC13259 was assumed to bind C8 subunits as a monomer. We employed AF2 (via the AlphaPullDown pipeline, v2.0.0) to assess *in silico* the likelihood of LIC13259 orthologs binding to each subunit of C8. Although AF has been used to model cross-kingdom interactions (28, 29), it can produce false-negative results or low scores due to lack of intermolecular covariance signal in the host-pathogen scenario (30). As such, we sampled 50 models (rather than the default 5) per combination of LIC13259 binding to each C8 subunit (in cows, humans, rats, and hamsters) to increase the probability of producing a confident model. We assessed the results using weighted ipTM + pTM score (Fig. 2A and 2B and Fig. S2A) and rescored the top model per species combination with pDockQ (Fig. S2B).

AlphaFold2-multimer outputs two confidence metrics for each predicted complex: pTM (predicted TM-score), which reflects overall model confidence, and ipTM (interface predicted TM-score), which specifically assesses the confidence of the predicted protein-protein interface; the weighted composite ipTM + pTM (0.8 × ipTM + 0.2 × pTM) is the standard summary metric used to evaluate predicted interactions. When assessing the results of AF multimer predictions, weighted ipTM + pTM scores >0.8 suggest high-quality, confident predictions; scores of 0.6–0.8 are often successful models worth exploring further; and scores <0.6 usually indicate failed predictions (31). However, in large-scale screens, weighted ipTM + pTM thresholds as low as 0.3 might be employed (32). The results suggested that the γ subunit was the most likely target of LIC13259 because, although the median score was lower, multiple higher confidence models emerged for *L. interrogans* LIC13259 binding to bovine C8γ, with the best quality model having a weighted ipTM + pTM score of 0.64 and pDockQ score of 0.36 (with a cut-off >0.23 sufficient to ensure model quality) (33, 34). Similar patterns of emergent higher quality structures were observed for *L. borgpetersenii* and *L. wolffii* binding bovine C8γ*,* though their maximum weighted ipTM + pTM scores fell below the 0.6 confidence threshold at 0.52 and 0.45, respectively. In contrast, the results for bovine C8α and C8β had a tight and consistent distribution of low scores, suggesting negative results. Human C8 subunit predictions were generally lower confidence; however, although not to the extent of the bovine screen, increased ipTM + pTM scores were observed for LIC13259 orthologs binding the γ subunit compared to the α and β subunits.

We then employed binding ELISAs to validate the *in silico* results for human and bovine C8 (Fig. 2C and D), which were in reasonable agreement with the computational screen predictions (Fig. 2A and B). Our results identified that LIC13259 of all *Leptospira* strains displayed greater binding specifically to the C8γ subunit, with P1 strains binding the most and the S1 strain binding the least. In contrast to *in silico* results, the P1 species *L. borgpetersenii* LIC13259 was suggested to have greater binding, instead of *L. interrogans. L. biflexa* LIC13259 displayed minimal binding to C8γ, with a fivefold decrease in binding compared to *L. borgpetersenii* LIC13259 (*P* < 0.0001). Human and bovine C8γ have a sequence identity of 84.4% and no host differences were observed for binding to LIC13259 proteins. LIC13259 protein from *L. borgpetersenii*, *L. interrogans*, and *L. biflexa* demonstrated minimal/no binding to either human or bovine C8β subunit (82.5% sequence identity), with the P2 species binding significantly more compared to *L. biflexa*. In contrast to *in silico* results, the experimental data also showed C8α binding. P2 species displayed significantly more binding of LIC13259 to C8α compared to *L. biflexa* (*P* < 0.0001). Regarding P1 species binding to C8α, a statistical difference was observed exclusively for *L. borgpetersenii* LIC13259 binding to human C8α (*P* < 0.0001). Human and bovine C8α have a sequence identity of 76.3%. Although *L. borgpetersenii* infects both humans and cattle, this finding suggests that the host immune response to the same pathogen differs, supporting the concept of host-specific adaptability.

### A short N-terminal loop drives LIC13259 binding

**Fig 3:**
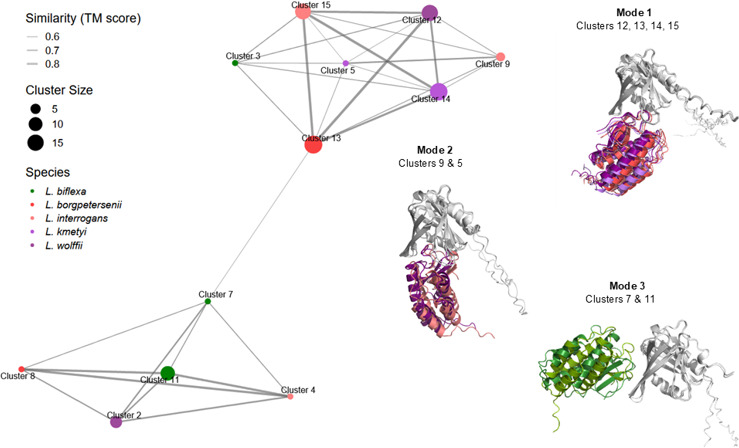
Multimer clustering revealed distinct binding modes for LIC13259 attaching to C8γ. The 25 structures per *Leptospira* LIC13259 binding to C8γ were reduced to clusters using Foldseek. Each cluster had a representative structure. Pairwise similarity between the representative structures was carried out, with the results visualized as a network with the Fruchterman-Reingold layout. Each node represents a cluster (with representative structure) determined by Foldseek; the size of the node is proportionate to the size of the cluster, and the color of the node corresponds to which *Leptospira* species the LIC13259 came from. Clusters with two or fewer members are not shown. Each edge indicates the structural similarity (determined by TM-score) between the representative clusters, with line weight proportionate to the TM-score. An edge was only included if the pairwise TM-score was >0.5 (35). Right, the representative structures for clusters overlaid to demonstrate the binding modes: two from the pathogenic module (Modes 1 and 2) and one from the non-pathogenic module (Mode 3).

Having established that LIC13259 binds to C8γ, we sought to characterize the P1 binding mode. Due to low confidence of initial predictions and recognizing the difficulties of host-pathogen prediction, we employed clustering approaches to gather information across all predictions. We clustered the binding poses per LIC13259 ortholog binding to C8γ based on structural similarity, producing overall representative structures per cluster. Pairwise structural similarity was then determined for the representative structures of LIC13259 orthologs binding to C8γ, with results visualized as a network (Fig. 3). This resulted in two distinct modules, the first containing the largest clusters from P1 and P2 species and the second with the largest cluster representing the saprophyte. These two communities corresponded to binding poses that either blocked the C8α-γ binding site (pathogenic; Modes 1 and 2) or low-confidence poses predicted in association with the exterior of C8γ (saprophyte; Mode 3).

**Fig 4:**
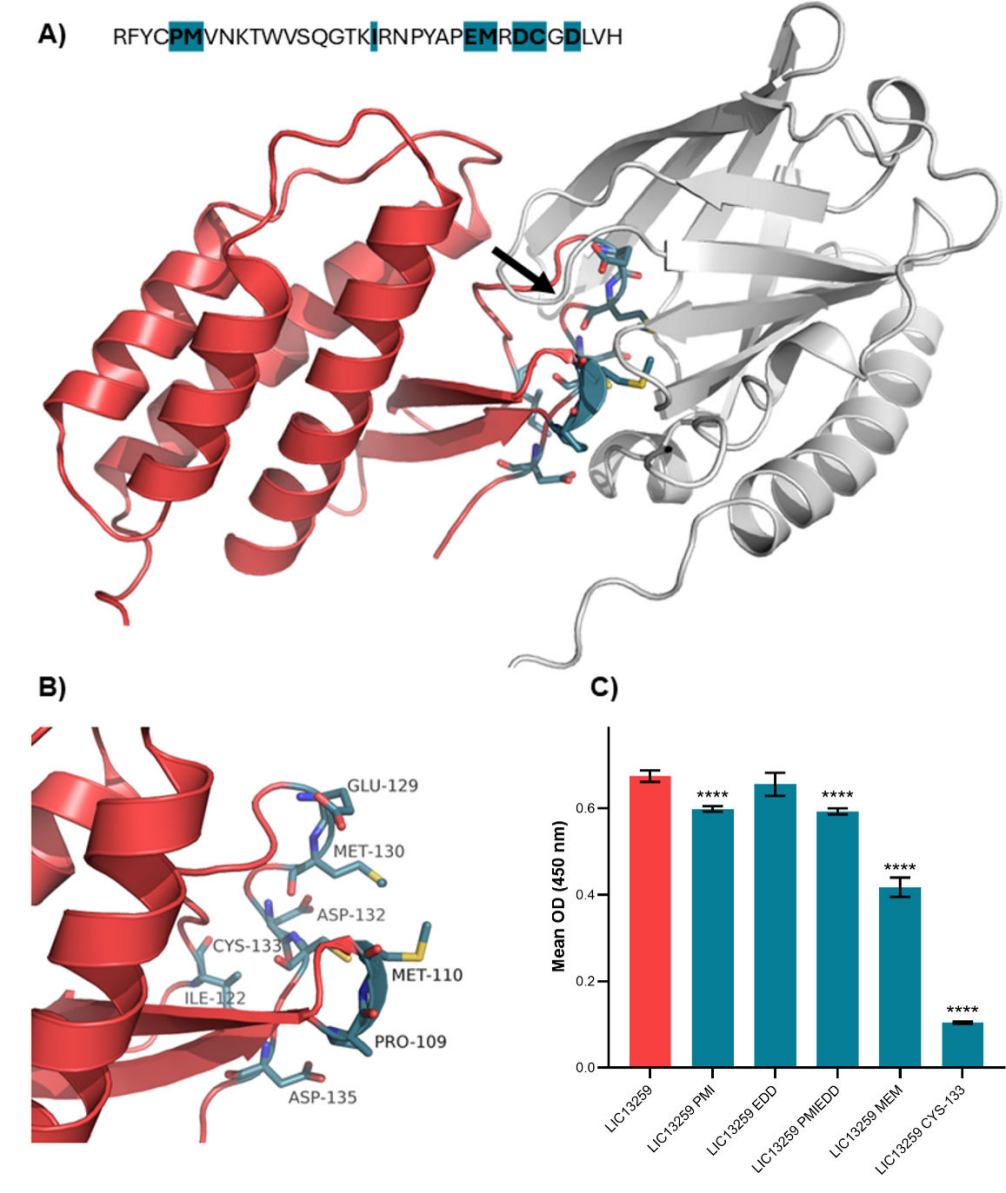
Mutations within the N-terminal loop of LIC13259 can restrict attachment to C8γ. (**A**) Cartoon representation of the highest quality binding pose between *L. interrogans* LIC13259 (red) and C8γ (gray). The binding loop, shown in teal, is indicated with an arrow, with residues of interest shown in stick representations. (**B**) Zoomed-in view showing residues most frequently involved in contacts at the interface. Those labeled and highlighted in teal were targeted for mutagenesis with alanine residues. Per-residue confidence scores are shown in Fig. S3. (**C**) Binding of recombinant protein *L. borgpetersenii* LIC13259 wild-type and mutants: PMI (PRO-109, MET-110, and ILE-122), EDD (GLU-129, ASP-132, and ASP-135), PMIEDD (PRO-109, MET-110, ILE-122, GLU-129, ASP-132, and ASP-135), MEM (MET-110, GLU-129, and MET-130), and CYS-133 to human C8 was qualitatively assessed by ELISA. Human C8 (5 µg/mL) was coated onto 96-well plates, and binding of recombinant LIC13259 proteins (12 µM) was measured by determining the optical density (OD) at 450 nm. Data are presented as *N* = 3 ± SD. Data were statistically analyzed using a one-way ANOVA followed by Dunnett’s multiple comparisons *post hoc* test (****P < 0.0001 compared to LIC13259).

Cluster inspection revealed that an N-terminal hairpin structure and short loop were consistently involved in contacts between LIC13259 and C8γ in Modes 1 and 2 (Fig. 4A) and were also where MSA identified sites of interest (Fig. 1). We determined which residues were most frequently involved in interface contacts and targeted these for mutagenesis and functional characterization (Fig. 4B and Fig. S3). Four mutants, with amino acid residues mutated to alanine, significantly inhibited attachment to C8γ: PMI (PRO-109, MET-110, and ILE-122), PMIEDD (PRO-109, MET-110, ILE-122, GLU-129, ASP-132, and ASP-135), MEM (MET-110, GLU-129, and MET-130), and CYS-133 (Fig. 4C). Mutation of the cysteine residue caused a dramatic decrease in the binding of LIC13259 to C8γ, so we took this residue forward for further analysis. Thermal stability assays confirmed that mutations did not impact protein stability (Fig. S4).

### LIC13259 binds to C8γ via a disulfide bond

**Fig 5:**
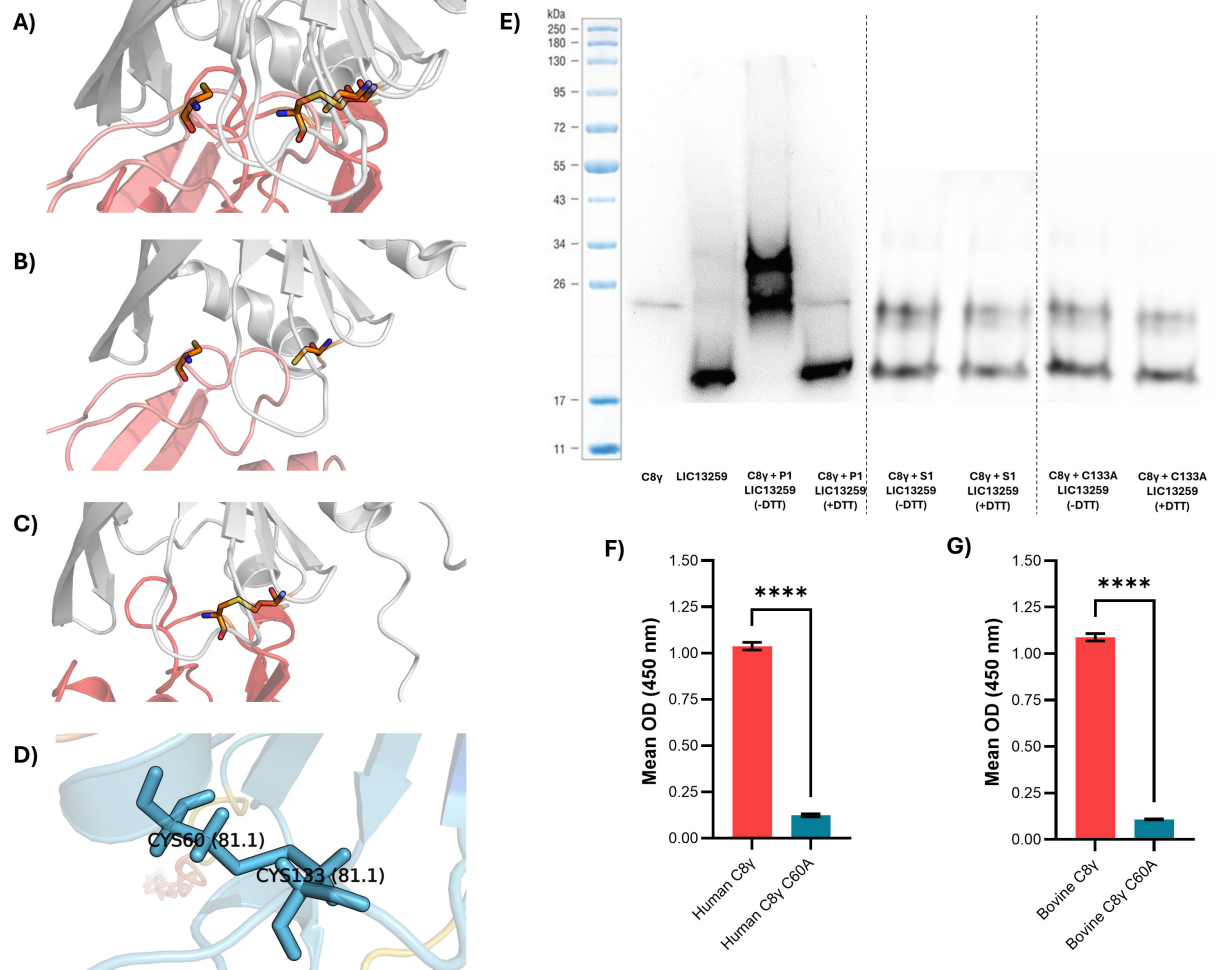
LIC13259 and C8γ bind via a disulfide bond. (**A**) The two P1 binding modes superimposed over each other. LIC13259 is shown in red, and C8γ is shown in gray. C8γ was used as a reference for the structural alignment. Cysteines are shown in orange as stick representations. (**B**) Mode 1, the more common but lower-quality predicted binding pose. (**C**) Mode 2, the higher-quality binding pose, with a predicted disulfide bond between LIC13259 CYS-133 and C8γ CYS-60 shown in yellow. (**D**) Zoomed-in view of the predicted disulfide, colored and annotated by quality score (pLDDT). (**E**) Recombinant *L. borgpetersenii* (P1), *L. biflexa* (S1) or C133A mutant LIC13259 proteins and human C8γ were incubated at 37°C for 1 h in the presence or absence of dithiothreitol (DTT, 50 mM). In the presence of DTT, disulfide bonds cannot form. Samples were run under non-reducing SDS-PAGE conditions and visualized by Western blotting. Standard protein ladder (11-250 kDa). Binding of *L. borgpetersenii* LIC13259 to human C8γ and human C8γ C60A (CYS-60 alanine substitution) (**F**) or bovine C8γ and bovine C8γ C60A (CYS-60 alanine substitution) (**G**) was qualitatively assessed by ELISA. Purified recombinant C8γ subunits or C8γ C60A mutants (5 µg/mL) were coated onto 96-well plates, and binding of recombinant LIC13259 protein (12 µM) was measured by determining the optical density (OD) at 450 nm. Data are presented as *N* = 3 ± SD. Data were statistically analyzed using unpaired *t*-tests (****P < 0.0001).

Clustering analysis revealed two distinct LIC13259 binding modes to C8γ for P1 and P2 species. While Mode 1 included larger clusters with broader sampling, Mode 2—though represented by fewer models—produced higher-quality predictions. In both conformations, the N-terminal loop of LIC13259 inserts into the hydrophobic pocket of C8γ. However, a critical distinction lies in the positioning of CYS-133. In Mode 1, CYS-133 is rotated away from C8γ’s CYS-60, whilst in Mode 2, these residues were in close enough proximity that a disulfide bond was predicted (Fig. 5A through D). The S-S distance of the disulfide was estimated to be ~2.07 Å (PISA), in keeping with expected disulfide geometry. The Mode 2 pose was verified using SDS-PAGE and western blotting to compare reducing and non-reducing conditions (dithiothreitol), which indicated LIC13259 from *L. borgpetersenii* binds C8γ via a disulfide bond (Fig. 5E).

Using gene synthesis for targeted mutagenesis to generate novel variants together with ELISA, it was validated that C8γ CYS-60 drives attachment to LIC13259 (Fig. 5F and G). Substituting CYS-60 with alanine (C60A) in both human and bovine C8γ significantly reduced LIC13259 binding (*P* < 0.0001), consistent with a covalent disulfide bond between LIC13259 CYS-133 and C8γ CYS-60 as the critical interaction mediating attachment. Despite all LIC13259 sequences containing CYS-133, our results suggest a statistically significant reduction in *L. biflexa* LIC13259 binding to C8γ (Fig. 2C and D). Sequence alignments revealed that surrounding residues of CYS-133 differ consistently between P1, P2, and S1 subclades (Fig. 1), suggesting that local sequence context is essential to bring CYS-133 into contact with CYS-60. Moreover, analyzing the protein by Western blotting after SDS-PAGE with and without DTT failed to demonstrate a disulfide bond between LIC13259 from *L. biflexa* or the LIC13259 CYS-133 mutant to C8γ (Fig. 5E).

**Fig 6:**
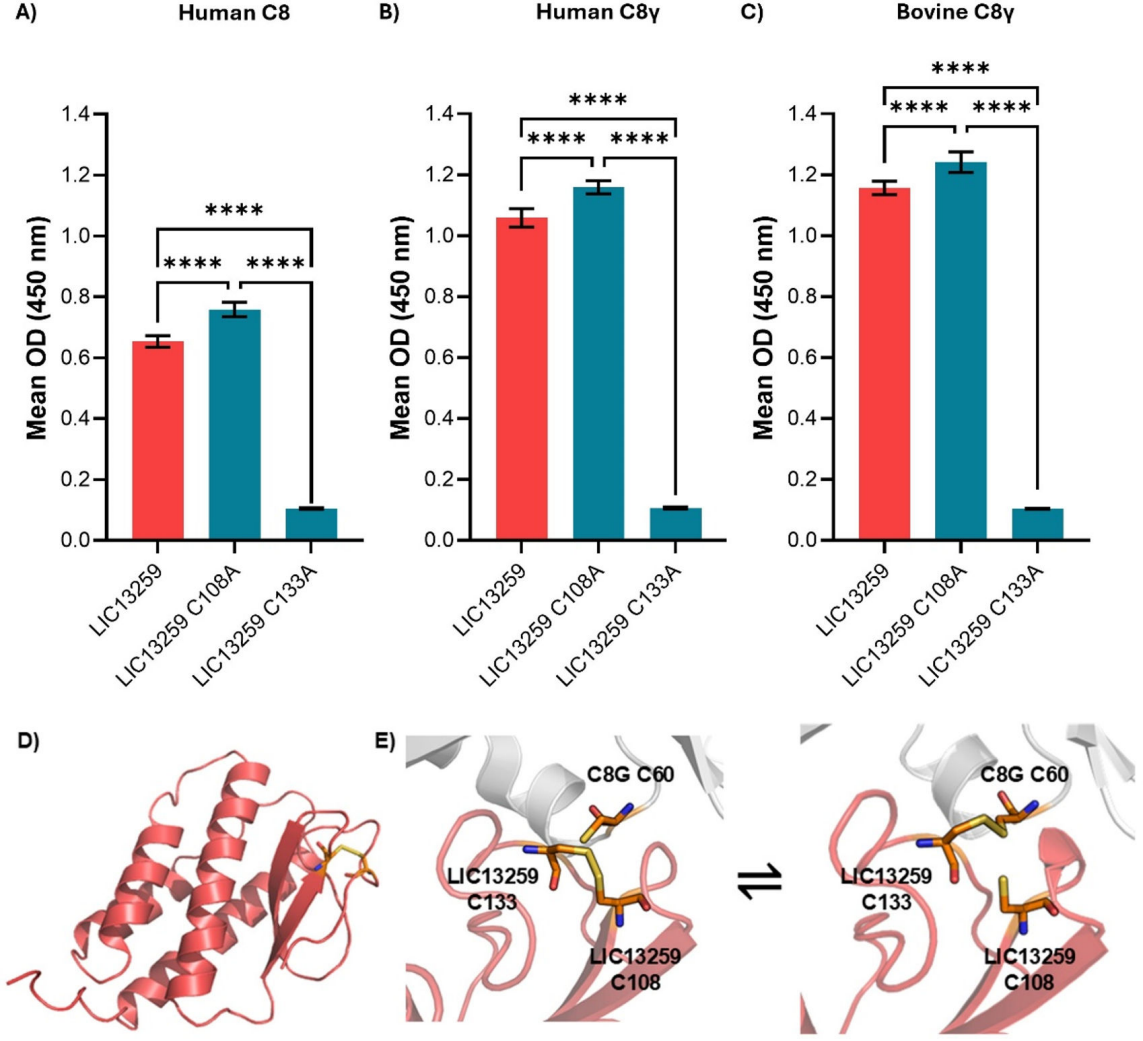
Impact of interrupting the LIC13259 CYS-108–CYS-133 intra-disulfide bond on C8 binding. Binding of the *L. borgpetersenii* LIC13259 mutant C108A and C133A to human C8 (**A**), human C8γ (**B**), and bovine C8γ (**C**) was qualitatively assessed by ELISA. Human C8 or purified recombinant C8γ subunits (5 µg/mL) were coated onto 96-well plates, and binding of recombinant LIC13259 proteins (12 µM) was measured by determining the optical density (OD) at 450 nm. Data are presented as *N* = 3 ± SD. Data were statistically analyzed using a one-way ANOVA followed by Tukey’s multiple comparisons *post hoc* test (****P < 0.0001). (**D**) LIC13259 structure showing the intra-chain disulfide between C108 and C133 as sticks. (**E**) Position of C8γ (C8G) C60 relative to the LIC13259 intra-chain disulfide and thiol-disulfide exchange at the interface.

Interestingly, monomeric models of LIC13259 were predicted to have an intra-chain disulfide bond between CYS-108 and CYS-133, which was absent in the multimeric LIC13259-C8γ complex. To further investigate the role of CYS-108 and CYS-133 in driving attachment to C8γ, recombinant *L. borgpetersenii* LIC13259 was generated with alanine substitutions (C108A and C133A). Restricted attachment of LIC13259 C133A to whole human C8 (Fig. 6A), human (Fig. 6B), and bovine (Fig. 6C) C8γ subunits was demonstrated by ELISA. LIC13259 C133A was found to ablate binding to whole human C8 (*P* < 0.0001), human C8γ (*P* < 0.001), and bovine C8γ (*P* < 0.0001) compared to the wild-type *L. borgpetersenii* LIC13259. Interestingly and importantly, LIC13259 C108A significantly increased binding to human C8, human C8γ, and bovine C8γ (*P* < 0.0001) compared to wild-type *L. borgpetersenii* LIC13259. Structural visualization of the intra-chain disulfide bond between CYS-108 and CYS-133 (Fig. 6D) and the relative binding position of C8γ reveals the close proximity of the three cysteine residues (Fig. 6E). Together, these data suggest that thiol-disulfide exchange occurs at the interface, whereby the deprotonated CYS-60 (thiolate) of C8γ attacks the internal disulfide bond between CYS-108 and CYS-133, forming a new intermolecular disulfide bond between C8γ CYS-60 and LIC13259 CYS-133. Where CYS-108 is absent from LIC13259, CYS-133 may be more readily accessible for disulfide bond formation, explaining the elevated binding. The disulfide swap involved in wild-type LIC13259 binding would then become the formation of a new intermolecular disulfide bond, which would be favored by the oxidizing nature of the extracellular environment.

Redox-mediated thiol-disulfide exchange has been observed in pathogenic bacteria as a strategy for surviving oxidative stress. *Escherichia coli* redox-regulated transcriptional activator RclR contains cysteine residues that are highly sensitive to oxidation by reactive chlorine species, which results in increased expression of genes required to survive the reactive chlorine species oxidative stress (36). In leptospires, previous research has described two glutaredoxins (disulfide oxidoreductases), *Lin*1CGrx and *Lin*2CGrx, identified in *L. interrogans* (37). Although it is evident that bacteria utilize thiol-disulfide exchange to resist the host’s immune response oxidative burst, it has yet to be reported as a mechanism for evasion of the complement system nor as a mechanism for cross-kingdom disulfide formation.

### LIC13259 from P1 species disrupts C8α binding to C8γ

**Fig 7:**
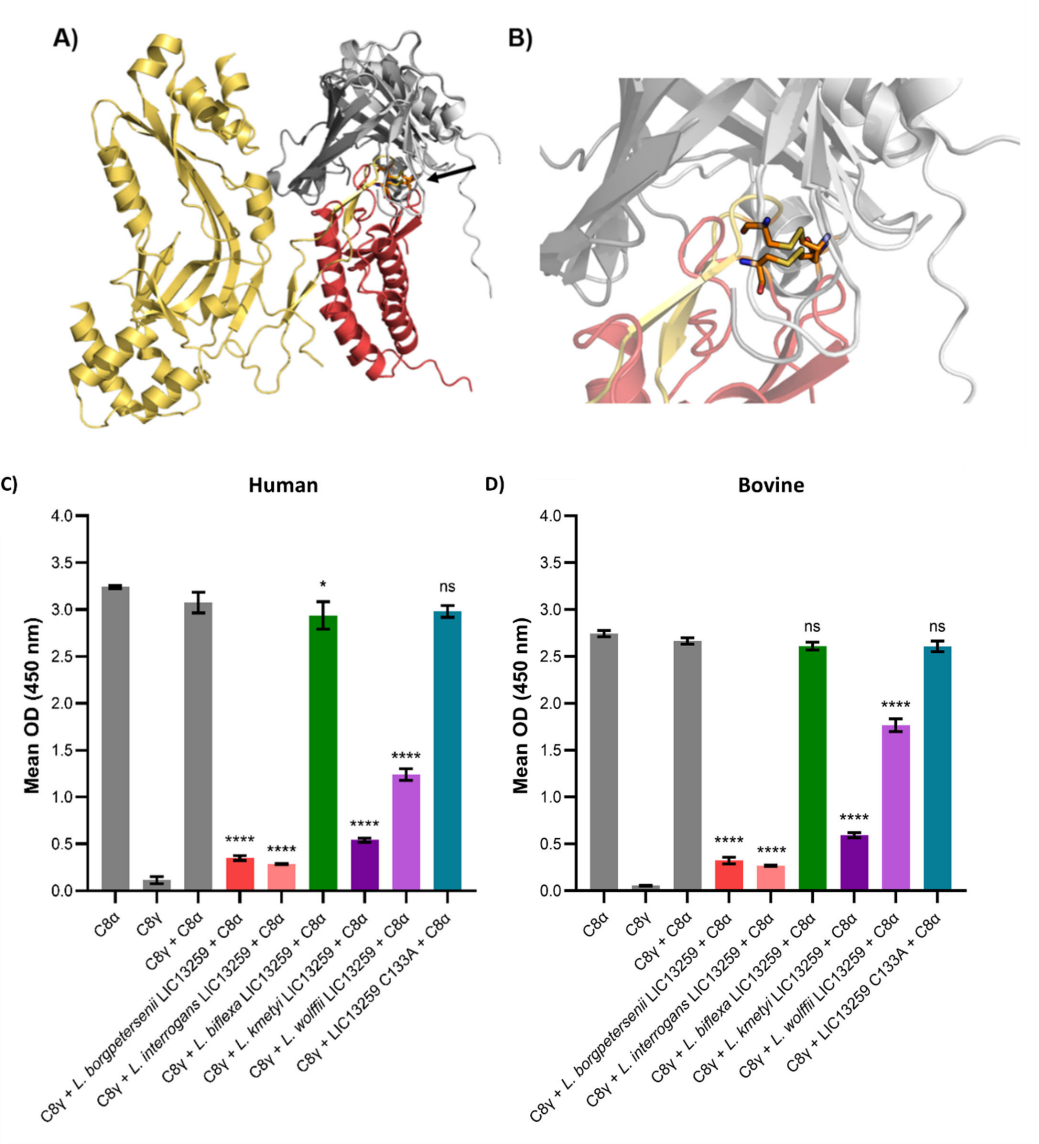
*L. borgpetersenii* and *L. interrogans* LIC13259 block C8α binding to C8γ. (**A**) The structure of C8α (yellow) and LIC13259 (red) in complex with C8γ (gray). The two structures were superimposed by aligning the C8γ subunits. The structure for the C8α-C8γ multimer was obtained from the PDB (accession: 2RD7). (**B**) Zoomed-in view showing the alignment of the C8α-C8γ disulfide bond and predicted disulfide bond between LIC13259 and C8γ. ELISAs were used to determine whether LIC13259 prevents C8α binding to C8γ for both human (**C**) and bovine (**D**) C8 subunits. Purified recombinant C8γ subunits (5 µg/mL) were coated onto 96-well plates followed by incubations with either *L. borgpetersenii*, *L. interrogans*, *L. kmetyi*, *L. wolffii*, *L. biflexa,* or *L. borgpetersenii* C133A recombinant LIC13259 proteins (12 µM), and subsequently the corresponding host C8α subunit (5 µg/mL). C8α subunit binding was determined by measuring the optical density (OD) at 450 nm. Data are presented as *N* = 3 ± SD. Data were statistically analyzed using a one-way ANOVA followed by Dunnett’s multiple comparisons *post hoc* test (****P < 0.0001; *P < 0.05; ns = not significant compared to C8γ + C8α).

C8 is composed of a disulfide-linked C8α-γ heterodimer and non-covalently associated C8β subunit (19–22). Notably, C8γ CYS-60 also forms a covalent linkage with C8α. Our results suggest that LIC13259 competes for the C8α binding pocket in C8γ, inhibiting formation of the MAC by co-opting the disulfide bond-forming CYS-60. Structural overlays of the known C8α-C8γ heterodimer (PDB: 2RD7) with the predicted LIC13259-C8γ complex showed that although the structure and orientation of LIC13259 greatly differ from C8α, the binding loop occupies the same location as the C8α hairpin, with both cysteines in near-identical conformations (Fig. 7A and B).

To validate that LIC13259 disrupts C8α binding to C8γ, ELISAs were completed by coating plates with human (Fig. 7C) or bovine (Fig. 7D) C8γ subunits, followed by incubations with either *L. borgpetersenii*, *L. interrogans*, *L. biflexa*, *L. kmetyi*, *L. wolffii*, or *L. borgpetersenii* C133A recombinant LIC13259 proteins, and subsequently the C8α subunit. Binding of *L. borgpetersenii* and *L. interrogans* LIC13259 before incubation with the C8α subunit significantly reduced C8α binding compared to C8α binding directly to C8γ (*P* < 0.0001). Similar C8α binding was observed to the P2 species *L. kmetyi* and *L. wolffii*. Conversely, less significant reduction of binding was evident when incubated with *L. biflexa* LIC13259 to human C8α (*P* < 0.05) and no significance to bovine C8α. There was no significant difference found between C8α subunit binding to C8γ compared to C8α binding in the presence of LIC13259 C133A.

Computational predictions and western blotting indicate that while P1 LIC13259 binds via a disulfide bond within the hydrophobic pocket of C8γ, S1 LIC13259 does not form a disulfide bond with C8γ and either associates peripherally or not at all (no significant scores were obtained for any individual models, Fig. 2B), leaving CYS-60 accessible for C8α binding. Thus, the P1 strains’ ability to block the C8α-C8γ disulfide bond underlies their capacity to reduce MAC formation and evade complement-mediated lysis.

### LIC13259 from P1 species reduces bactericidal activity

**Fig 8:**
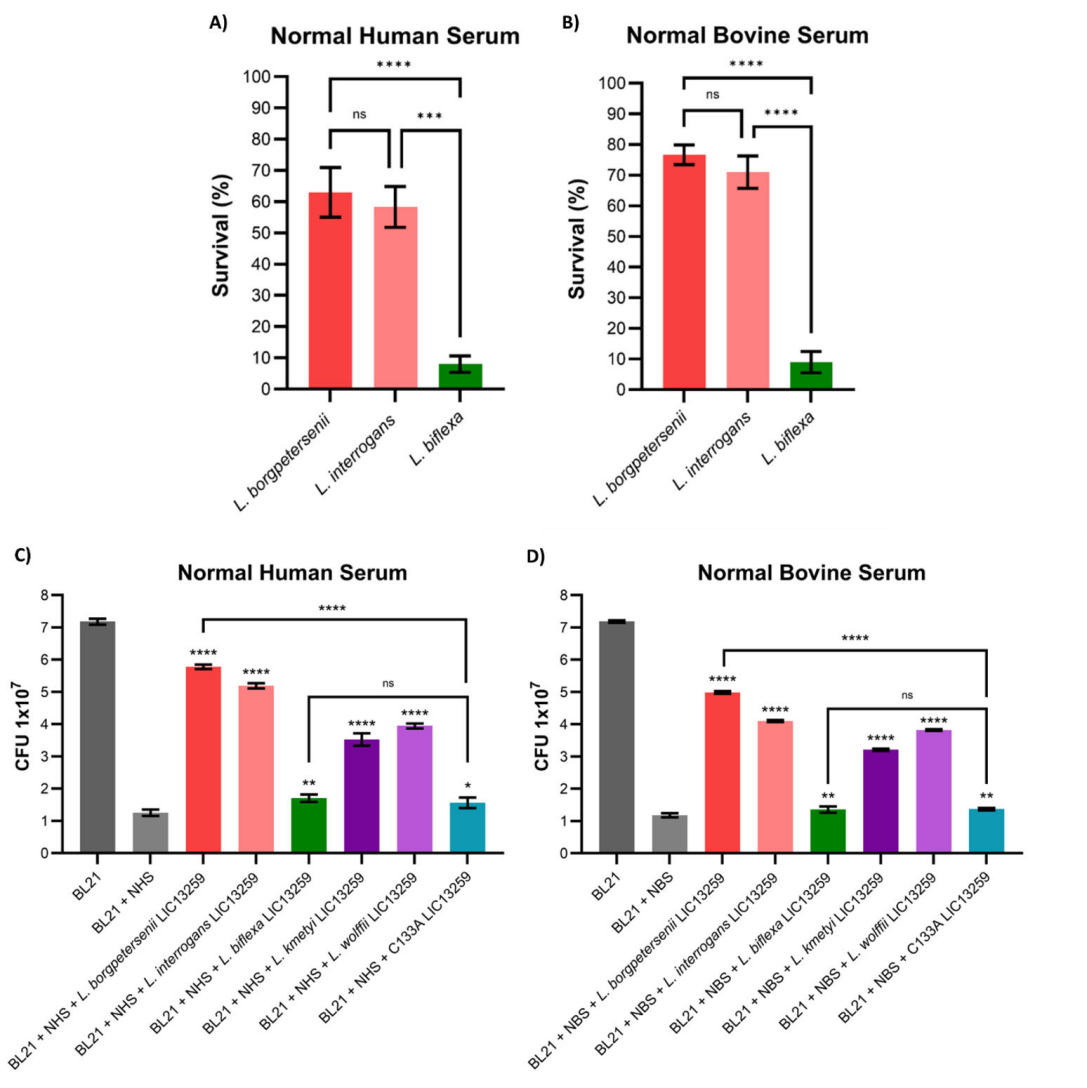
*Leptospira* serum susceptibility and inhibition of complement-mediated killing by LIC13259 from different *Leptospira* species and the C133A mutant. Serum susceptibility assay: 5 × 10^7^ cultured *Leptospira* species *L. borgpetersenii* serovar Hardjo type bovis (JB197), *L. interrogans* serovar Hardjo type Prajitno (Hardjoprajitno), and *L. biflexa* serovar Patoc (Patoc 1) were incubated in 40% serum for 2 h. Percentage survival was calculated by comparing the bacterial cell counts in normal versus heat-inactivated serum; human (**A**), bovine (**B**). *Leptospira* species incubated in inactivated serum displayed a 96–100% survival. Data are presented as *N* = 3 ± SD. Data were statistically analyzed using a one-way ANOVA followed by Tukey’s multiple comparisons *post hoc* test (****P < 0.0001; ***P < 0.001; and ns = not significant). Complement-mediated killing assay: 1.2 × 10^8^ *E. coli* (BL21) cells were incubated with 12 µM of either *L. borgpetersenii*, *L. interrogans*, *L. kmetyi*, *L. wolffii*, *L. biflexa*, or *L. borgpetersenii* C133A recombinant LIC13259, and either normal human serum (NHS) (**C**) or normal bovine serum (NBS) (**D**). *E. coli* survival was determined by calculating the CFU/mL. Data are presented as *N* = 3 ± SD. Data were statistically analyzed compared to the BL21-NHS/NBS control using a one-way ANOVA followed by Dunnett’s multiple comparisons *post hoc* test (****P < 0.0001; ***P < 0.001; **P < 0.01; and *P < 0.05). A two-tailed unpaired *t*-test was used to statistically analyze the differences between BL21 + NHS/NBS + *L. borgpetersenii* LIC13259 and BL21 + NHS/NBS + C133A LIC13259 (****P < 0.0001), as well as BL21 + NHS/NBS + *L. biflexa* LIC13259 and BL21 + NHS/NBS + C133A LIC13259 (ns = not significant).

Consistent with previous studies, our data show that in the presence of normal serum (human and bovine), pathogenic *Leptospira* are resistant, whereas saprophytic *L. biflexa* are susceptible to complement killing (Fig. 8A and B) (16, 38–40). No significant differences in survival were observed between P1 species in normal human and bovine serum, suggesting that the mechanisms are similarly effective. However, there was a statistically significant reduction in survival for S1 species *L. biflexa* compared to P1 species in normal human and bovine serum. *L. biflexa* displays a 7- to 9-fold decrease in survival compared to P1 species, with only 8–9% of *L. biflexa* surviving.

To directly assess the protective role of LIC13259 against complement-mediated bactericidal activity, complement-mediated killing assays were performed where *E. coli* BL21 cells were incubated with recombinant LIC13259 proteins and either human (Fig. 8C) or bovine (Fig. 8D) normal serum. The presence of normal serum significantly reduced the number of *E. coli* cells. In the presence of the P1 *L. borgpetersenii* and *L. interrogans* LIC13259 and serum, *E. coli* cell numbers were significantly greater (4.5-fold increase) compared to serum alone (*P* < 0.0001). P2 *L. kmetyi* and *L. wolffii* LIC13259 similarly induced a substantial significant 3-fold increase in *E. coli* cell numbers (*P* < 0.0001), albeit to a lesser extent compared to P1 LIC13259 proteins. *L. biflexa* (S1) LIC13259 only displayed a 1.3-fold increase of *P* < 0.01 in *E. coli* cell numbers. Comparatively, the *L. borgpetersenii* LIC13259 C133A mutant had a much lower increase in *E. coli* cell numbers (survival) (only 1.25-fold increase), which was significantly different compared to wild-type LIC13259 (*P* < 0.0001), and did not exhibit a statistically significant change compared with *L. biflexa* LIC13259. This suggests that preventing P1 LIC13259 binding to C8γ reduces complement-mediated killing to the same degree as incubation with S1 LIC13259.

**Fig 9:**
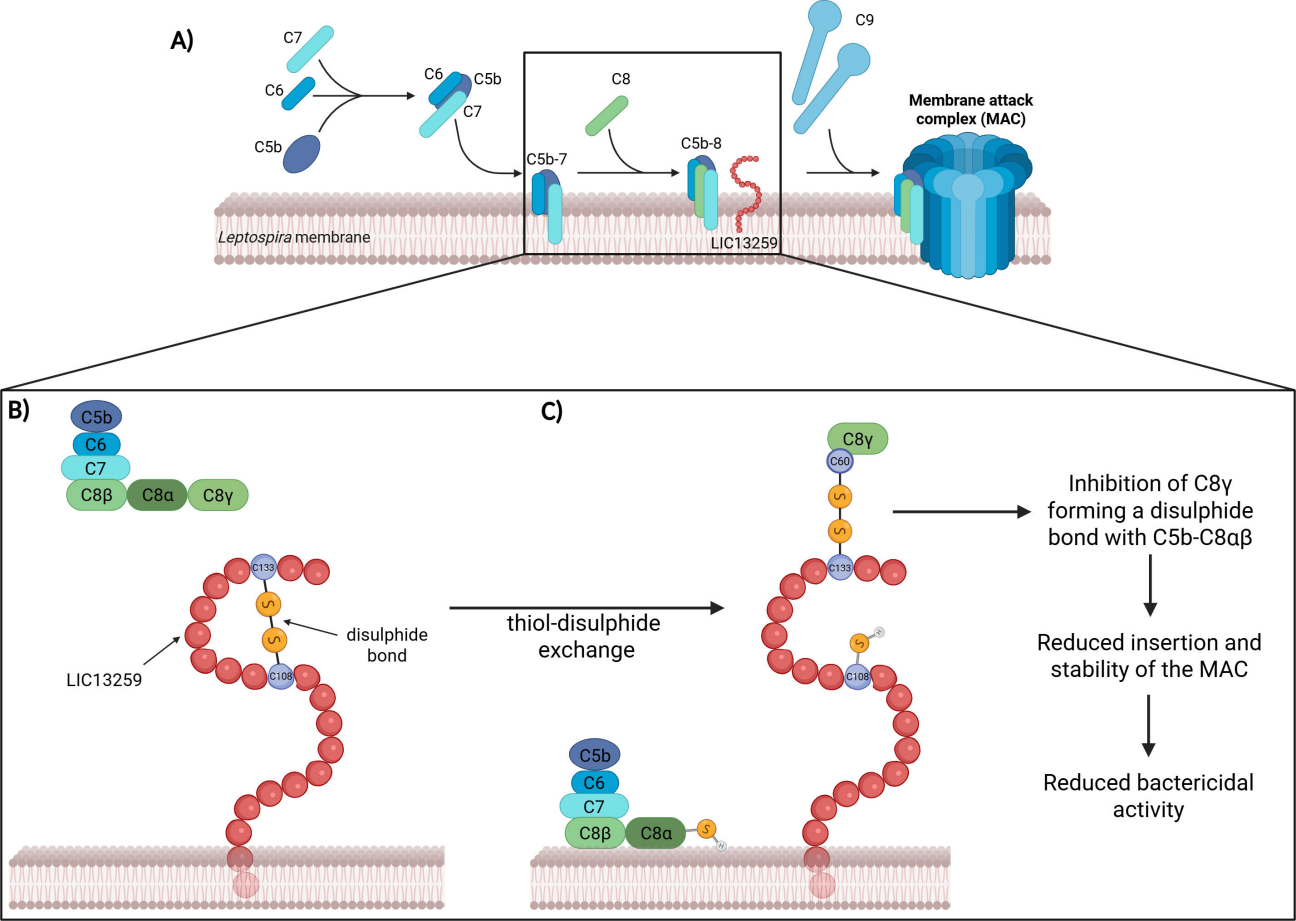
The leptospire P1 LIC13259 Binding Mechanism to C8γ. (**A**) The membrane attack complex (MAC) is part of the complement innate immune response against invading pathogens and is formed on the surface of bacterial membranes. Complement components C5b, C6, and C7 associate, undergoing a conformational change allowing for the binding of C8 for insertion into the bacterial membrane. C9 molecules bind to the C5b-8 complex, forming a pore structure (MAC), resulting in the influx of fluid and subsequent bacterial cell lysis. (**B**) C8 is composed of a disulfide-linked C8α-γ heterodimer and non-covalently associated C8β subunit. The C8β subunit binds to the complex, allowing C8α-γ to insert into the lipid membrane via C8α binding to C8β. P1 *Leptospira* express LIC13259 on the surface, which contains an intra-disulfide bond between cysteine (**C**) 133 and 108. (**C**) During bacterial infections, reactive oxygen species create a redox environment that promotes disulfide bond rearrangement. By thiol-disulfide exchange, the intra-disulfide bond between C108 and C133 undergoes nucleophile attack by the free thiol (SH) group of C8γ C60, resulting in the formation of a new disulfide bond between LIC13259 C133 and C8γ C60. Therefore, C5b-C8αβ is inserted into the membrane without association of C8γ, reducing the stability of the MAC in the *Leptospira* membrane and reducing bactericidal activity. Figure created in BioRender.

In summary, these data indicate LIC13259 from P1 strains evade complement killing by disrupting C8α-C8γ binding, reducing MAC formation. We propose that complement evasion by LIC13259 is mediated through a redox-driven thiol-disulfide exchange mechanism that disrupts the structural integrity of C8 during MAC assembly. In circulation, C8 exists as a disulfide-linked C8α–C8γ heterodimer associated with C8β; however, during infection, the dynamic extracellular oxidizing/redox-active environment generated by reactive oxygen species can promote disulfide bond rearrangement. Increasing evidence demonstrates that extracellular proteins are subject to thiol modifications under inflammatory conditions, including thiol-disulfide exchange, which can regulate immune processes and pathogen interactions (41). We hypothesize that, in this context, the intramolecular disulfide bond between CYS-108 and CYS-133 in LIC13259 is first reduced, consistent with the behavior of allosteric redox-active disulfides that undergo reduction to induce functional changes distal to their active sites (42, 43). Liberation of CYS-133 would then enable nucleophilic attack on the CYS-60–CYS-197 disulfide bond in the C8γ-C8α heterodimer, forming a new inter-kingdom intermolecular disulfide linkage between LIC13259 and C8γ, competitively displacing C8α. Structural studies support the feasibility of this interaction, showing that C8γ becomes accessible during MAC assembly and that C8 undergoes substantial conformational rearrangements that expose previously buried regions (44, 45). As a result, C8γ is sequestered away from the C8αβ complex, allowing membrane insertion of C5b–C8αβ but impairing proper MAC stabilization (22). Although C8γ is not strictly required for lytic activity, its absence reduces MAC stability and bactericidal efficiency, thereby permitting survival of P1 Leptospira. This model is further supported by parallels in bacterial and viral systems, where host-mediated disulfide reduction and exchange are essential for virulence factor activation and host cell entry, often catalyzed by surface oxidoreductases such as protein disulfide isomerase (46, 47). In contrast, LIC13259 from non-pathogenic *L. biflexa* does not engage in this redox interaction with C8γ, preserving C8α–C8γ association and rendering the bacteria susceptible to complement-mediated killing. The mechanism is summarized in Fig. 9.

To identify differences in *Leptospira* interactions between hosts; human and bovine responses were compared throughout this study. Although subtle differences were observed, overall trends remained the same, indicating conserved susceptibility across species. However, the pathogenic *Leptospira* strains selected have a similar disease phenotype in both human and bovine hosts. Further probes of host specificity involving analysis of more hosts (e.g., canine) and diverse P1 strains that are host-restricted (e.g., *L. borgpetersenii* serovar Ballum has been reported more prevalent in rodents compared to cattle [48]) may reveal further host-specific immune evasion strategies.

The ability of leptospires to evade host immunity through inhibition of the complement system has been reported. However, the specific molecular mechanisms by which this occurs and its relation to *Leptospira* strain pathogenicity was previously unexplored. Our study reveals a novel and biologically significant mechanism by which *Leptospira* pathogenicity is mediated via a cross-kingdom thiol-disulfide exchange. This interaction represents a previously uncharacterized disulfide-linked host-pathogen protein complex, in which the bacterial protein LIC13259 forms a disulfide bond with complement component C8γ. To the best of our knowledge, this is the first report of a disulfide bond mediating a host-pathogen PPI across kingdoms, suggesting a novel evolutionary strategy to evade innate immune surveillance. Due to the experimental validation of AF2 predictions, we consider the structural model validated.

In conclusion, these findings add a new dimension to our understanding of host-pathogen interactions, where pathogens often hijack host processes via molecular mimicry (30). We propose that pathogenic *Leptospira* exploit this principle not just through surface complementarity but via a covalent cross-species mimicry interface with LIC13259. This work not only advances our understanding of *Leptospira* pathogenesis but expands the arsenal of mechanisms by which prokaryotic pathogens might evade the eukaryotic immune response, contributing a novel example to the broader field of host-pathogen interaction biology. Future research into whether similar cross-kingdom disulfide-mediated mimicry mechanisms exist in other bacterial species may open new avenues towards understanding pathogenesis across species and underpin future therapeutics and vaccines.

## MATERIALS AND METHODS

### Computational screening

C8 and LIC13259 sequences were obtained from UniProt (49) and the NCBI database (50), respectively. MSAs were created using MMseqs2 (51). ColabFold was used to predict individual structures for LIC13259 orthologs and C8 subunits using default parameters (52), as well as to produce models for multimeric C8 in complex with LIC13259 (*n* = 50). AlphaPullDown (53), a Python package built around AlphaFold-Multimer (26), was employed in “Pulldown” mode to predict structures of LIC13259 orthologs bound to individual C8 subunits. Default parameters were used, except for the number of models (*n* = 50) and the inclusion of a relaxation step. A Python script was used to remove any models that exhibited backbone clashes. Models were assessed using weighted ipTM + pTM score (0.8 iPTM + 0.2 PTM) (33), pDockQ score (54), and, where relevant, pLDDT scores. The 25 most confident models per combination of LIC13259 and C8 were subjected to multimer clustering using Foldseek (55) to determine pairwise structural similarity for deriving representative structures of LIC13259 orthologs binding to C8γ. Resultant TM-scores were visualized as a network, with the Louvain method used to identify communities. Interface residues involved in regular contacts across the models were identified using Gemmi (56). Python was used to process the AF2 models, with R statistical programming environment (version 4.2.3) (57) used for downstream analysis and visualization of *in silico* results, unless stated otherwise. PISA was used to assess disulfide bond lengths and interfaces for the top-quality models (58).

### Phylogenetic analysis

LIC13259 sequences were aligned and phylogenetic trees created using MEGA 6.06 (59) with bootstrapping.

### Recombinant LIC13259 protein purification

LIC13259 proteins were expressed using pET-28a(+) vectors, purchased from Twist Bioscience (sequences in Table S2). Expression vectors were transformed into BL21-AI One Shot *E. coli* competent cells (Invitrogen, C607003) and starter cultures produced by inoculating a single transformed colony into LB medium with kanamycin (50 µg/mL) overnight at 37°C and 200 rpm. Inoculates were diluted and cultures grown at 37°C and 200 rpm until an OD_600_ of 0.6 was reached. Protein expression was induced by 1 mM IPTG and 0.2% l-arabinose, followed by overnight incubation at 20°C and 120 rpm.

Based on the protocol described in Kamaruzaman et al. (60), cells were harvested by centrifugation and lysed with 50 mg lysozyme (Sigma Aldrich, 62971). Suspensions were sonicated (Soniprep-150, MSE) for 5 min with a cycle of 20 s on and 10 s off at 45% amplitude. Soluble material was harvested, proteins purified using Ni-NTA agarose (Qiagen, 30210), and eluted using imidazole (250 mM). Imidazole was removed by overnight dialysis using 10 mm dialysis membranes (Sigma Aldrich, D9277) in PBS at 4°C. Dialyzed proteins were filtered (0.22 µM) and stored at −80°C. Protein concentration was determined before and after freezing using a Qubit fluorometer (Invitrogen, 45259-425) and protein assay kit (Invitrogen, Q33211). Thermal stability assays confirmed that mutations did not impact protein stability or structural integrity (Fig. S4). Additionally, no evidence of precipitation or signs of protein instability were observed, and protein integrity was confirmed by SDS-PAGE (method described in supplementary information).

### Recombinant C8 subunit protein purification

Recombinant C8 subunits (sequences in Table S3) were expressed as described for rLIC13259 proteins with exceptions described in the supplementary information.

### ELISA

Immobilon 2HB 96-well plates (3455) were coated with 100 µL of either human C8 (Complement Technology, A125) or rC8 subunits diluted in PBS at a final concentration of 5 µg/mL. Plates were incubated at 37°C for 1 h, then 4°C overnight, washed three times with PBS plus 0.5% Tween-20 (Sigma Aldrich, P1379), and incubated at 37°C for 1 h in 300 µL of blocking buffer (1% BSA [Sigma Aldrich, A8022] in PBS). Washing was repeated, and 100 µL of either rLIC13259 or rC8α at 12 µM were added to the wells, followed by 1 h incubation at 37°C. Wells were washed and incubated in 100 µL of primary antibody (1:2,000) for 1 h at 37°C (mouse anti-polyhistidine [Merck, H1029] for rLIC13259; rabbit anti-C8alpha [Amsbio, A05720] for rC8α). Washing was repeated, and 100 µL of either anti-mouse or anti-rabbit peroxidase antibody (Sigma, A0412; Invitrogen, 31460, 1:10,000) was added to the wells, followed by incubation at 37 °C for 1h. Wells were washed and incubated for 10–20 min with 100 µL TMB substrate (Interchim, UP664781). The reaction was stopped using 100 µL of 0.5 M HCl. Absorbance was measured at a wavelength of 450 nm using an AMG Labtech plate reader. Gelatin (Sigma, G1890) was used to coat plate wells as a negative control.

### Bacterial culture

*Leptospira* species *L. borgpetersenii* (JB197), *L. interrogans* (Hardjoprajitno), and *L. biflexa* (Patoc 1) purchased from Amsterdam UMC leptospirosis reference center were cultured in Difco *Leptospira* medium base EMJH (BD, 279410) and 10% Difco *Leptospira* enrichment EMJH (BD, 279510) at 30°C and passaged weekly. *E. coli* BL-21 (DE3) cells (Invitrogen, C600003) were cultured in LB broth overnight at 37°C and 200 rpm.

### *Leptospira* serum susceptibility assay

As outlined by Isaac and Barbosa (61), cultured leptospires were harvested by centrifugation, washed in PBS, and resuspended in 1 mL PBS. 5 × 10^7^ *Leptospira* cells were incubated in 40% normal or heat-inactivated (56°C for 30 min) serum for 2 h at 37°C. Viable leptospire cell count was determined using phase contrast microscopy and a Petroff-Hausser counting chamber. Normal complement-certified human (Jackson ImmunoResearch, 009-000-121) and bovine (Gibco, 16170086) sera were used.

### Complement-mediated killing assay

As described by Kumar et al*.* (62), rLIC13259 proteins (12 µM) were incubated with either 10% normal human or bovine serum for 30 min at 37°C. 1.2 × 10^8^ *E. coli* cells were incubated for 30 min at 37°C in serum/rLIC13259. Samples were plated on LB agar plates, incubated overnight at 37°C, and the CFU/mL calculated.

### Statistical analysis

Data were analyzed using GraphPad Prism 10.4.1 and statistically analyzed using either a one- or two-way ANOVA followed by either a Tukey’s or Dunnett’s multiple comparisons *post hoc* test or unpaired *t*-tests. A value of *P* ≤ 0.05 was considered a statistically significant difference. Experiments were conducted in duplicate or triplicate (technical repeat), and the *N* represents the number of biological repeats.

## Data Availability

The authors declare that all relevant data supporting the findings of this study are available within the paper (and its Supplemental material files). Additional data set information of this study is available from the corresponding author upon reasonable request. All code used to perform the analyses in this study is available at https://github.com/CBFLivUni/Leptospira-LIC13259-C8G. The repository contains the bash scripts, Quarto markdown documents, and supporting scripts required to reproduce the results, starting from two provided FASTA files. Installation and set-up instructions are provided in the repository README. The license is an MIT License, and the doi is https://doi.org/10.5281/zenodo.16903768.

## SUPPLEMENTAL MATERIAL

Supplemental material mbio.00878-26-s0001.pdf Additional materials and methods, Fig. S1 to S5, and Tables S1 to S4.

Raw data mbio.00878-26-s0002.xlsx Raw data and expanded statistical analysis for Fig. 2, 4 to 8, and S4.

ASM does not own the copyrights to Supplemental Material that may be linked to, or accessed through, an article. The authors have granted ASM a non-exclusive, world-wide license to publish the Supplemental Material files. Please contact the corresponding author directly for reuse.
