# Supplementary material for: AlphaFold reveals how pathogenic Leptospira use cross-kingdom thiol-disulfide exchange to evade the complement membrane attack complex
Source: mBio. 2026 Jun 15;17(7):e00878-26. doi: 10.1128/mbio.00878-26 (PMC13344006; doi:10.1128/mbio.00878-26)
Supplement: Supplemental material — Additional materials and methods, Fig. S1 to S5, and Tables S1 to S4. [file mbio.00878-26-s0001.pdf]

Supplementary Information

Materials and Methods

SDS-PAGE and Western blotting

Recombinant LIC13259 proteins and human C8γ proteins were incubated in sample buffer (1M Tris-HCl pH 6.8, 50% glycerol, 0.25% bromophenol blue in distilled water) for 1h at 37°C in the presence or absence of dithiothreitol (50mM). Samples and ladder (11-250 kDa; New England Biolabs, P7718S) were loaded onto 12% Mini-PROTEAN TGX protein gels (Biorad, 4561043) and blotted onto PVDF membranes using a BioRad Trans Blot Turbo Transfer System. Detection used 1:3000 mouse anti-polyhistidine (Merck, H1029) as primary antibody and 1:10,000 anti-mouse peroxidase (Sigma, A0412) as secondary antibody with SuperSignalTM Wet Pico Plus Chemiluminescence Substrate (Thermo Scientific, 34580) and an Azure Biosystems chemiSOLO Imager (CS1000).

Recombinant C8 subunit protein purification exceptions

After expression induction, cells were incubated for 3h at 37°C and 200rpm, then centrifuged at 5000xg for 15min at 4°C and stored at -20°C overnight. Cell lysis buffer: 20mM Tris-HCl (pH 7.9), 500mM NaCl, 2mM dithiothreitol (DTT, Fluorochem, M02712), 50mg lysozyme. To selectively solubilise and remove contaminants from insoluble pellet, the pellet was resuspended in 150mL of wash buffer (20mM Tris-HCl (pH 7.9), 500mM NaCl, 2mM DTT, 1M urea, 1% Triton® X-100 (Fisher, BP151)) and stirred for 1h at room temperature (RT). Insoluble material harvested by centrifugation at 10,000xg for 30min at 4°C and subsequently solubilised for 16h, gently rolling at RT, in 15mL of buffer containing 20mM Tris-HCl (pH 7.9), 500mM NaCl, 2mM DTT, 8M urea. Remaining insoluble material was removed by centrifugation at 10,000xg for 30min at 4°C. Solubilised proteins were sterilised using a 0.45µM filter and then diluted 50X in refolding buffer (20mM Tris-HCl (pH 7.9), 500mM NaCl, 2mM DTT) in a dropwise manner (0.65mL/min) with continuous stirring at RT, then stirring was continued for a further 2h. Equilibration buffer (10mL): 20mM Tris-HCl (pH 7.9), 150mM NaCl, 2mM DTT. Wash buffer (25mL): 20mM Tris-HCl (pH 7.9), 150mM NaCl, 2mM DTT, 20mM. Elution buffer: 20mM Tris-HCl (pH 7.9), 150mM NaCl, 2mM DTT, 250mM imidazole. Eluted rC8 subunits were dialysed overnight at 4°C with gently stirring in 10mm dialysis membranes in distilled water with 20mM Tris-HCl (pH 7.9) and 150mM NaCl to remove imidazole and DTT. Removal of the his-tag from the rC8 subunits was achieved using TEV protease (NEB, P8112) following manufacturer's instructions. Thermal stability assays confirmed that mutations did not impact protein stability or structural integrity (Figure S4). Additionally, no evidence of precipitation or signs of protein instability were observed, and protein integrity confirmed by SDS-PAGE.

Thermal stability assay

GloMelt™ (Biotium, BT33021-T) was used following manufacturer's instructions with final rLIC13259 protein concentrations at 1.5µg/uL. Melt curves were measured using a Bio-Rad

CFX Opus 96 Real-Time PCR System (12011319). Temperature was initially held at 20°C for 30s, increasing 0.05°C/s, to a maximum temperature of 99°C. Melting temperatures were determined using the R statistical programming environment (version 4.4.3)[50] to compute the positive derivative of the fluorescence intensity with respect to per unit increase in temperature (dF/dT) from the relative fluorescent unit (RFU) calculated in the Bio-Rad CFX Maestro Software. Results confirmed the alanine substitutions did not alter the protein structures when compared to the wild type (Figure S4).

Figures

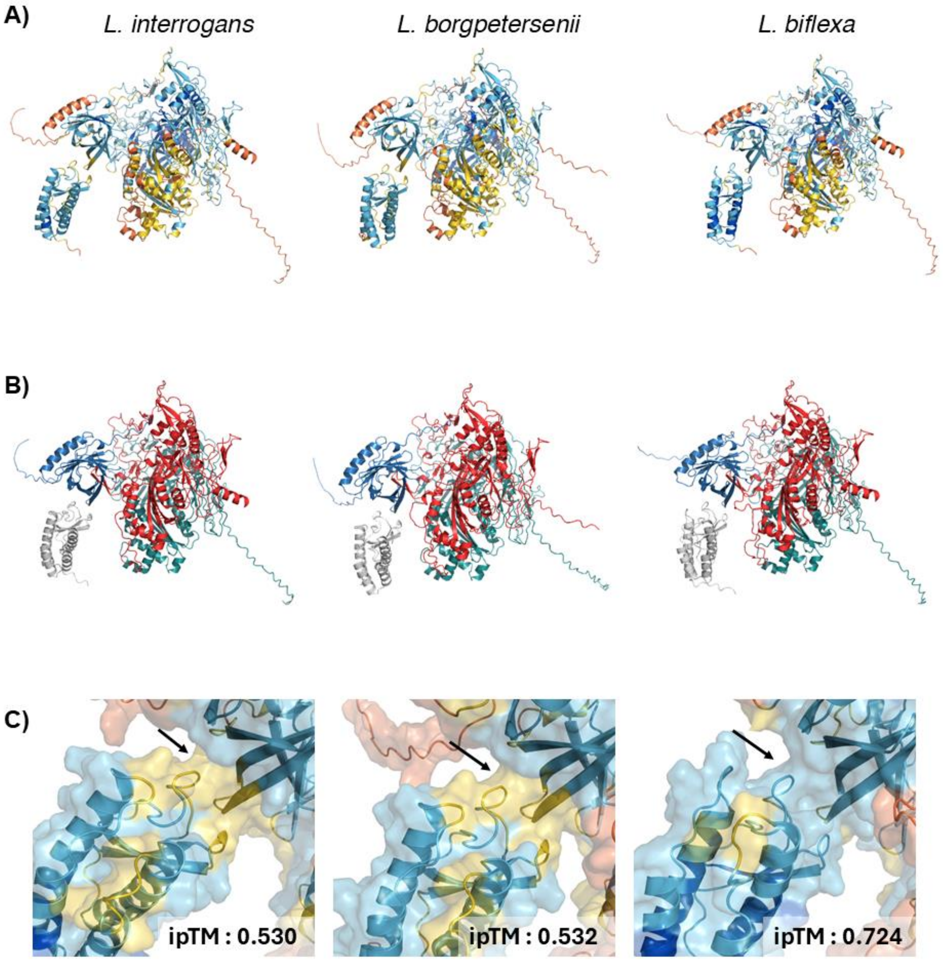

Figure S1: AlphaFold assessment of LIC13259 binding to fully assembled C8. A) Representative AlphaFold structural models of LIC13259 bound to the C8 complex for *L. interrogans, L. borgpetersenii, and L. biflexa*. For each species, the model with the highest ipTM score for the LIC13259–C8γ interface was selected. Structures are shown in cartoon representation coloured by per-residue confidence (pLDDT): >90 (dark blue), very high confidence; 70–90 (light blue), confident; 50–70 (yellow), low confidence; <50 (orange), very low confidence, often indicating flexible or disordered regions. (B) The same structures as in (A), coloured by subunit. LIC13259 is shown in grey, while C8 subunits are coloured as C8α (red), C8β (teal), C8γ (blue). (C) Zoomed in cartoon view of the predicted interface between LIC13259 and C8 for the models, as shown in (A). A transparent representation of the protein surface is overlaid. The interface is indicated with an arrow and each panel is labelled with its corresponding ipTM score. It should be noted that in all structures LIC13259 forms a very small interface (Table S1) on the periphery of the structure. It can also be observed that the overall confidence of the interfaces (as determined by the pLDDT scores) is quite low despite the overall high iPTM scores.

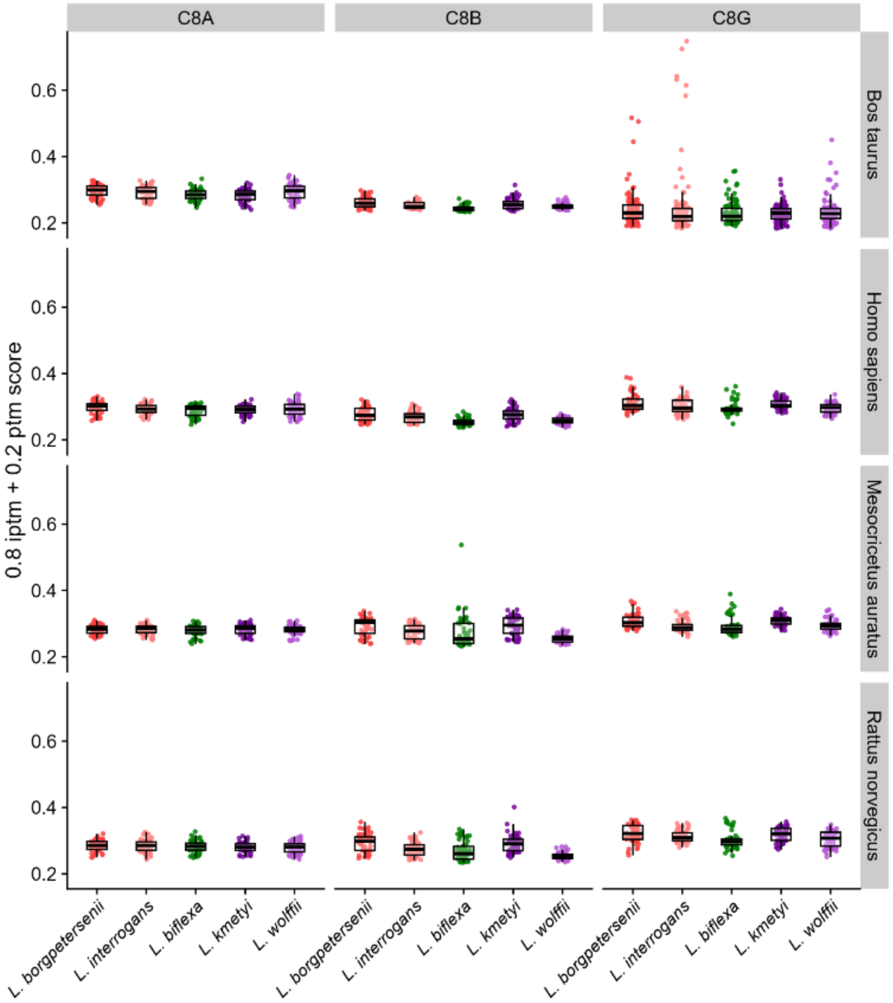

Figure S2A: *In silico* modelling of LIC13259 binding to hamster and rat C8. Box plots showing the distribution of 0.8 iPTM + 0.2 PTM confidence scores for the predicted multimers between *Leptospira* LIC13259 orthologues and hamster or rat C8 subunits. Each individual point represents a model (n=50). Points are coloured by species, with pathogenic species are shown in red, intermediate species shown in purple, and saprophytic species shown in green. The centre line is the median value, the box limits are the upper and lower quartiles, and the whiskers are 1.5 × the interquartile range.

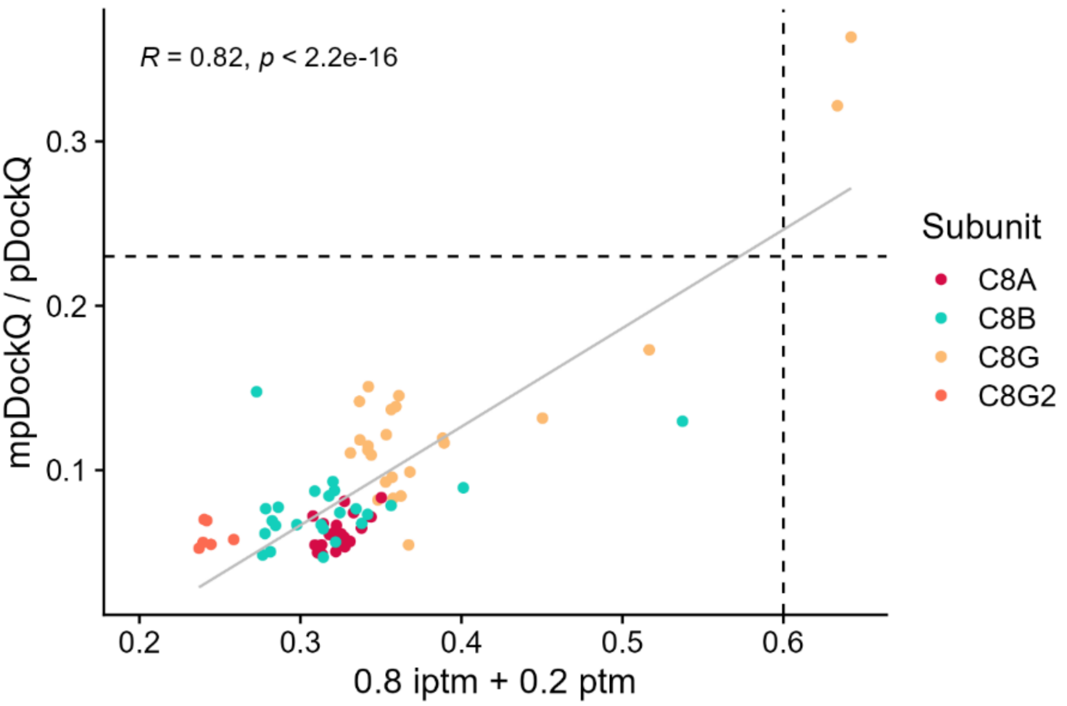

Figure S2B: Correlation between 0.8 ipTM + 0.2 pTM scores and mpDockQ scores. Scores were both calculated by AlphaPullDown for the top pose per combination of LIC13259 orthologues binding to C8 subunits. Each point is top binding pose; they are coloured by the receptor C8 subunit. Only hamsters possess the C8G2 subunit. The dotted lines represent thresholds for confident binding poses (0.23 for mpDockQ and 0.6 for ipTM+PTM score).

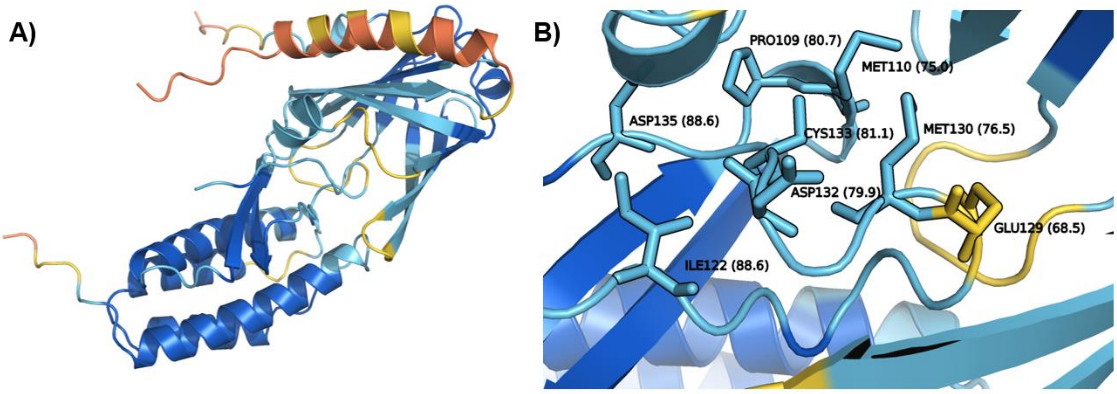

Figure S3: Local model quality and interface residues of the predicted *L. interrogans* LIC13259/C8γ complex. A) Structural model coloured by pLDDT confidence scores provided by AlphaPullDown. Colours indicate model reliability: >90 (dark blue) very high confidence; 70–90 (light blue) confident; 50–70 (yellow) low confidence; <50 (orange) very low confidence, often indicating flexible or disordered regions. **B**) Close-up view of the predicted protein–protein interface highlighting residues at the interaction surface. Residues are labelled with their corresponding pLDDT scores. These interface residues were selected as targets for site-directed mutagenesis in subsequent functional analyses to assess their role in mediating the LIC13259–C8γ interaction.

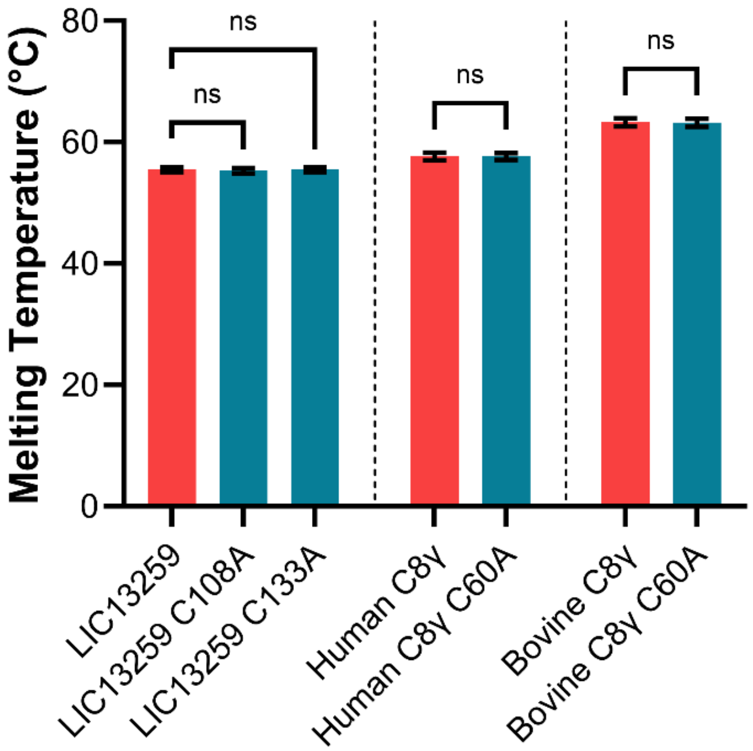

Figure S4: Thermal stability assay of the LIC13259 and C8γ alanine substitution mutants. Melt curves of the recombinant *L. borgpetersenii* LIC13259, human C8γ and bovine C8γ proteins and the mutants with an alanine substitution at CYS-108 (C108A), CYS-133 (C133A), CYS-60 (C60A) was determined using a GloMelt™ thermal stability assay. The melting temperature was determined using the R statistical programming environment to compute the positive derivative of the fluorescence intensity with respect to per unit increase in temperature (dF/dT) from the relative fluorescent unit (RFU) calculated in the Bio-Rad CFX Maestro Software. Data is presented as N=3±SD. Data was statistically analysed using two-tailed unpaired t tests (ns=not significant).

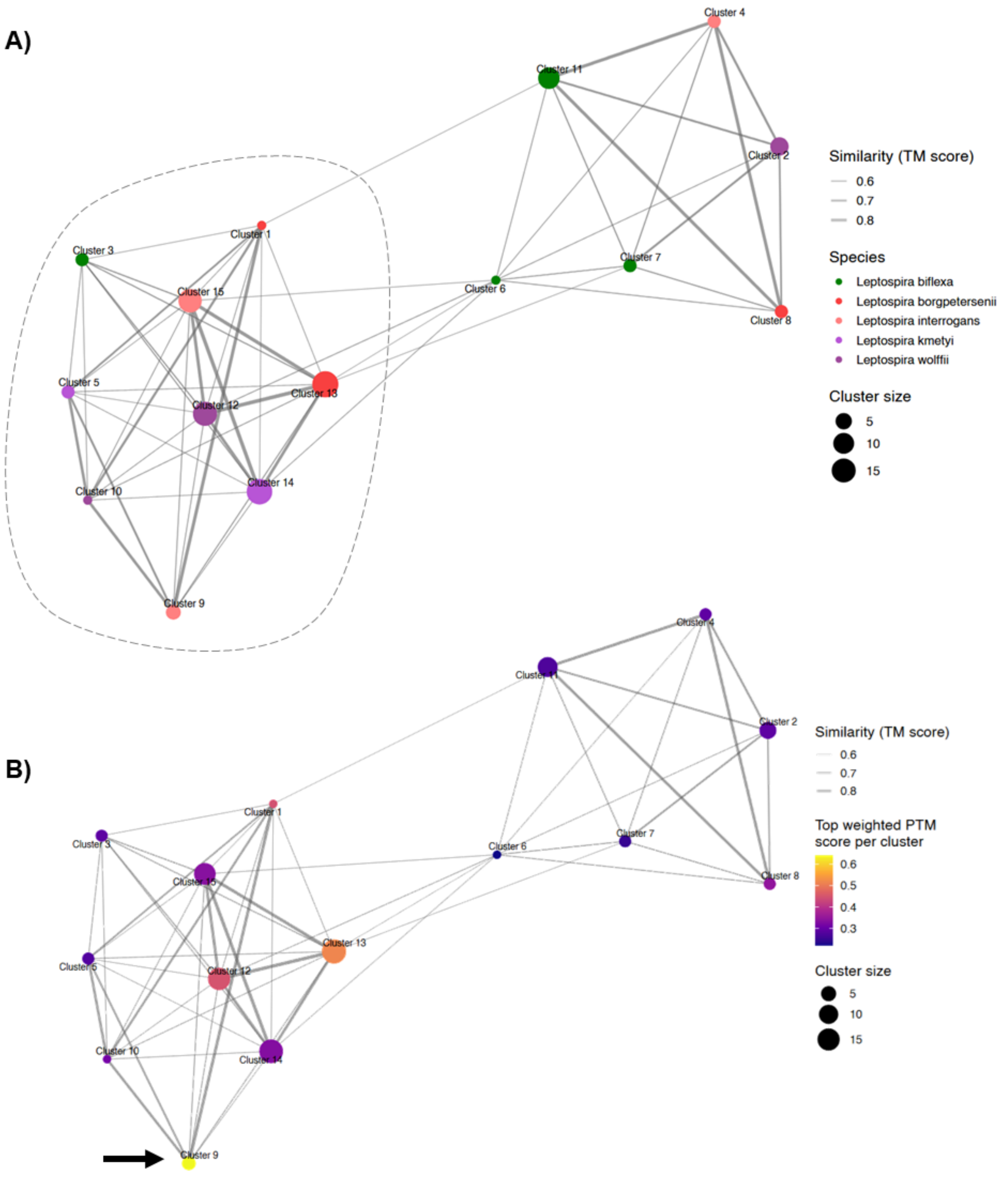

Figure S5: LIC13259/C8γ bovine multimer clustering network. A) The 25 structures per Leptospira LIC13259 binding to C8γ were reduced to clusters using Foldseek. Each cluster had a representative structure. Pairwise similarity between the representative structures was carried out, with the results visualised as a network with the Fruchterman-Reingold layout. Each node represents a cluster (with representative structure) determined by Foldseek; the size of the node is proportionate to the size of the cluster, and the colour of the node corresponds to which Leptospira species the LIC13259 came from. Smaller clusters were not filtered. Each edge indicates the structural similarity (determined by TM-score) between the representative clusters, with line weight proportionate to the TM-score. An edge was only included if the pairwise TM-score was >0.5. The pathogenic module is circled. B) The same network as (A) but instead coloured by AlphaFold2 0.8 ipTM + 0.2 pTM confidence scores. The highest ranked cluster containing the LIC13259-C8γ disulphide is highlighted.

Tables

Table S1: Comparison of LIC13259-C8γ interfaces in LIC13259-C8γ dimer vs LIC13259 in complex with a C8 multimer. For the multimer the model with the highest ipTM score for the LIC13259–C8γ interface was selected and compared against the best ranked LIC13259–C8γ model (determined by weighted PTM score). Interface parameters were generated using PISA.

	**LIC13259–C8γ** *(L. interrogans)*	**Full C8 multimer (C8γ interface)** *(L. interrogans)*
**Buried surface area**	~1,157 Ų	~256 Ų
**Interface residues (LIC13259)**	27	10
**Interface residues (C8γ)**	31	12
**H-bonds**	14	2
**Salt bridges**	15	0

Table S2: Recombinant LIC13259 protein sequences.

Strain	Amino Acid Sequence of LIC13259
L. borgpetersenii	MGSYYHHHHHHLESTSLYKKAGSCQKTPLVITESEKDVLQQILVENESIHSFLMKEEGKIPDTSKLISHIKTLISLNGGLKSSAEK<br>MKNFLQDKDTKDIEKFFQAYSSFSENLADSLKLAGGTGVFNRFYCPMINKTWVSHGTKIQNPYAPEMRDCGDLVR*
L. interrogans	MGSYYHHHHHHLESTSLYKKAGSCKKPDPAVTEQEKDILQQILMENESIHKFLMKEEEKIPDTNQLIAHVVELVSLNGGLKSQ<br>AEKMQNSLKDKETQDVEKFFQAYSSFSENLAESLKLAGGTGVFNRFYCPMVNKTWVSQGTKIRNPYAPEMRDCGDLVH*
L. biflexa	MGSYYHHHHHHLESTSLYKKAGSCKEEVVPFGPQHQKLVETLLAGNQSVLEQFLKENPKPNWKEFSETIQSLVASDHPKLRS<br>WGEQILTHIPTDQTDLESSYEKISQIQEILIQIRSEVPNQLKYNRFYCPMVDKSWLMTGREVKNPYAPEMRDCGELMQ*
L. kmetyi	MGSYYHHHHHHLESTSLYKKAGSCKKAQPQITEAEKDALQRILVENESIHDFLMKEENRIPDINKLVARVQELSSLNGGLKESA<br>EKMGNSLKDKETKDVEKFFLAYSSFSENLAESLKLAGGTGVFNKFFCPMVNKTWVSHGTKIQNPYAPEMRDCGDLVP*
L. wolffii	MGSYYHHHHHHLESTSLYKKAGSCGKKENKPLLENEKALFESVLVENDKIVKSLLTTEEVAPDIKALSAALTALESAKGGLEEAA<br>IQMKKDLAATENSDAETAFKAYSKFSESLAIVMKERGLQSGRNRFYCPMVKKTWVLAGQKIQNPYAPDMRECGDLIP*
L. borgpetersenii C108A mutant	MGSYYHHHHHHLESTSLYKKAGSCQKTPLVITESEKDVLQQILVENESIHSFLMKEEGKIPDTSKLISHIKTLISLNGGLKSSAEK<br>MKNFLQDKDTKDIEKFFQAYSSFSENLADSLKLAGGTGVFNRFYAPMINKTWVSHGTKIQNPYAPEMRDCGDLVR*
L. borgpetersenii C133A mutant	MGSYYHHHHHHLESTSLYKKAGSCQKTPLVITESEKDVLQQILVENESIHSFLMKEEGKIPDTSKLISHIKTLISLNGGLKSSAEK<br>MKNFLQDKDTKDIEKFFQAYSSFSENLADSLKLAGGTGVFNRFYCPMINKTWVSHGTKIQNPYAPEMRDAGDLVR*
L. borgpetersenii PMI mutant	MGSYYHHHHHHLESTSLYKKAGSCQKTPLVITESEKDVLQQILVENESIHSFLMKEEGKIPDTSKLISHIKTLISLNGGLKSSAEK<br>MKNFLQDKDTKDIEKFFQAYSSFSENLADSLKLAGGTGVFNRFYCAAINKTWVSHGTKAQNPYAPEMRDCGDLVR*
L. borgpetersenii EDD mutant	MGSYYHHHHHHLESTSLYKKAGSCQKTPLVITESEKDVLQQILVENESIHSFLMKEEGKIPDTSKLISHIKTLISLNGGLKSSAEK<br>MKNFLQDKDTKDIEKFFQAYSSFSENLADSLKLAGGTGVFNRFYCPMINKTWVSHGTKIQNPYAPAMRACGALVR*
L. borgpetersenii PMIEDD mutant	MGSYYHHHHHHLESTSLYKKAGSCQKTPLVITESEKDVLQQILVENESIHSFLMKEEGKIPDTSKLISHIKTLISLNGGLKSSAEK<br>MKNFLQDKDTKDIEKFFQAYSSFSENLADSLKLAGGTGVFNRFYCPMINKTWVSHGTKIQNPYAPAMRACGALVR*
L. borgpetersenii MEM mutant	MGSYYHHHHHHLESTSLYKKAGSCQKTPLVITESEKDVLQQILVENESIHSFLMKEEGKIPDTSKLISHIKTLISLNGGLKSSAEK<br>MKNFLQDKDTKDIEKFFQAYSSFSENLADSLKLAGGTGVFNRFYCPAINKTWVSHGTKIQNPYAPAARDCGDLVR*

Table S3: Recombinant C8 subunit sequences.

Protein	Accession Number	Amino Acid Sequence
Human C8α	P07357	MGSYYHHHHHHENLYFQGQEKVNQRVRRAATPAAVTCQLSNWSEWTDCFPCQDKKYRHRSLLQPNKFGGTICS<br>GDIWDQASCSSSTTCVRQAQCGQDFQCKETGRCLKRHLVCNGDQDCLDGSDEDDCEDVRAIDEDCSQYEPIPGS<br>QKAALGYNILTQEDAQSVYDASYYGGQCETVYNGEWRELRYDSTCERLYYGDDEKYFRKPYNFLKYHFEALADTGIS<br>SEFYDNANDLLSKVKKDKSDSFGVTIGIGPAGSPLLVGVGVSHSQDTSFLNELNKYNEKKFIFTRIFTKVQTAHFKMR<br>KDDIMLDEGMLQSLMELPDQYNYGMYAKFINDYGTHYITSGSMGGIYEYILVIDKAKMESLGITSRDITTCFGGSLGI<br>QYEDKINVGGGLSGDHCKKFGGGKTERARKAMAVEDIISRVRGGSSGWSGGLAQNRSTITYRSWGRSLKYNPVVI

		DFEMQPIHEVLRHTSLGPLEAKRQNLRRALDQYLMEFNACRCGPCFNNGVPILEGTSCRCQCRLGSLGAACEQTQ<br>TEGAKADGSWSCWSSWSVCRAGIQERRRECDNPAPQNGGASCPGRKVQTQAC*
Human C8β	P07358	MGSYYHHHHHHENLYFQGERPHSFGSNAVNKSFAKSRQMRSVDVTLMPIDCELSSWSSWTTCDPCQKKRYRYAY<br>LLQPSQFHGEPCNFSDKEVEDCVTNRPCGSQVRCEGFVCAQTGRCVNRRLLCNGDNDCGDQSDEANCRRIYKKC<br>QHEMDQYWGIGSLASGINLFTNSFEGPVLDHRYYAGGCSPHYILNTRFRKPYNVESYTPQTQGKYEFILKEYESYSD<br>FERNVTEKMASKSGFSFGFKIPGIFELGISSQSDRGKHYIRRTKRFSHTKSVFLHARSDLEVAHYKLKPRSLMLHYEFL<br>QRVKRLPLEYSYGEYRDLFRDFGTHYITEAVLGGIYEYTLVMNKEAMERGDYTLNNVHACAKNDFKIGGAIEEVYVS<br>LGVSVGKCRGILNEIKDRNKRDTMVEDLVVLVRGGASEHITTLAYQELPTADLMQEWGDAVQYNPAIIKVKVEPLYE<br>LVTATDFAYSSTVRQNMKQALEEFQKEVSSCHCAPCQGNGVPVLKGSRCDCICPVGSQGLACEVSYRKNTPIDGKW<br>NCWSNWSSCSGRRKTRQRQCNNPPPQNGGSPCSGPASETLDCS*
Human C8γ	P07360	MGSYYHHHHHHENLYFQGQKPQRPRRPASPISTIQPKANFDAQQFAGTWLLVAVGSACRFLQEQGHRAEATTLH<br>VAPQGTAMAVSTFRKLDGICWQVRQLYGDTGVLGRFLLQARDARGAVHVVVAETDYQSFAVLYLERAGQLSVKLYA<br>RSLPVSDSVLSGFEQRVQEAHLTEDQIFYFPKYGFCEAADQFHVLDEVRR*
Human C8γ C60A	-	MGSYYHHHHHHENLYFQGQKPQRPRRPASPISTIQPKANFDAQQFAGTWLLVAVGSAARFLQEQGHRAEATTLH<br>VAPQGTAMAVSTFRKLDGICWQVRQLYGDTGVLGRFLLQARDARGAVHVVVAETDYQSFAVLYLERAGQLSVKLYA<br>RSLPVSDSVLSGFEQRVQEAHLTEDQIFYFPKYGFCEAADQFHVLDEVRR*
Bovine C8α	Q2KIH5	MGSYYHHHHHHENLYFQGIQEKVNWRVSRAVQSFTPTAVSCQLDNWAEWTDCFPCQDKKYRYRSLLQPNKFGG<br>TICSGNVWDQASCHSPTACLSQAQCGQDFQCKETGRCLKRHLVCNGDKDCLDGSDEDDCEDVRILENDCSQYDPI<br>PGSEKAALGYNILTQEEAQHVYDARYYGGQCETVYNGEWRELQYDPACERLYYGDDDKYFRKPYNFLKYHFEAQA<br>DTKISSEIYNDANDLLTKVKNDKSVSSGLTIGVGIRGVPVTVTAGVSMSQDAAFLKKLSKYHEKKYSFMRIFTKVQTA<br>HFKMRRENIVLDEGMLQSLMELPERYHYGMYAKFINDYGTHYITSGSMGGVYEYILVLNREKMETAGVTSAEIQKC<br>FGVSLGIEYEYSEAIQIKGSSSLGPCKKSGDGKLTENEKAMGVEDFISRVRGDSSGWGGSLTQDSSLVTYRSWGRSLK<br>YNPAVIDFEMKPIHEILQHTNLGSLETKRQNLRRALDKYLMEFNACRCGPCFNNGEPILEGTSCKCQCPVGHQGLA<br>CEQMQSEGAQADGRWSCWSSWSACRSGTQERRRECNNPAPQNGGASCPGHRVQTQAC*
Bovine C8β	F1N102	MGSYYHHHHHHENLYFQGVPSSRGERPPSLGSNAVNESLAKTRQTRSVDVTPLPIDCELSSWSSWTTCDPCQKKR<br>YRHAYLIRPSQFHGEQCNFSDKEVEDCVSNRPCRSQVRCEGFVCAQTGRCINRRLLCNGDNDCGDQSDEANCRKT<br>YKKCQQEMEQYWAIGSLASGINLFTNNLEGPVLDHRYYAGLCSPHYILNTRFRKPYNVENFIPQTRGKYDFTLTEYES<br>YSDFEHNVTGTAISRSSFSLGFKIAGIFELGYSSMSNIGQHFISRIRRFSHTKSKFLHARSDLEVAHYKLKTRNLMLHHE<br>FLQRVKQLPLEYSYGEYRDLFRDFGTHYITEAVLGGIYEYTLIMNKEAMDRGDYSLKNIHDCAKHDFKIGAAITSVYLK<br>LGVSEAKCSDILNEIKDRNKKDSMVKDLVVLVRGGASEYVTALAYKELPTADLMQEWGDAVQYNPDIIKVKVMPLY<br>ELVTATDFAYSSTVKQNMKKALEEFQKEVDSCQCAPCQGNGVPVLKGTRCECICPAGFQGSACEVTHRKNVPIDGK<br>WNCWSNWSPCSGGHKTRQRQCTNPPPGNGGNPCSGPASETLNC*
Bovine C8γ	A8YXZ2	MGSYYHHHHHHENLYFQGQRVRRPPRPPSPISTIQPKANFDAQQFSGTWLLVAVASPCRYLQEQGHRAEATTLHV<br>APQGSAMVVSTFRKLDGICWAVRQLYGDTGRPGRFLLQARGARGPVDVVVGDTDYRGFAVLYLERARQLSVKLYG<br>TSWGPGARVCCARRAVGWAGWALSSLPPAPALQPKCGDRTGRGSGAAGVRSQPEAHSPAPRAPCRPLPRGCPG<br>ARVSCTQSARSL*
Bovine C8γ C60A	-	MGSYYHHHHHHENLYFQGQRVRRPPRPPSPISTIQPKANFDAQQFSGTWLLVAVASPARYLQEQGHRAEATTLHV<br>APQGSAMVVSTFRKLDGICWAVRQLYGDTGRPGRFLLQARGARGPVDVVVGDTDYRGFAVLYLERARQLSVKLYG<br>TSWGPGARVCCARRAVGWAGWALSSLPPAPALQPKCGDRTGRGSGAAGVRSQPEAHSPAPRAPCRPLPRGCPG<br>ARVSCTQSARSL*

Table S4: Summary of bovine LIC13259-C8γ clusters. The 25 structures per Leptospira LIC13259 binding to C8γ were reduced to clusters using Foldseek. Each cluster had a representative structure. The table shows the cluster label (as shown in figure S5), cluster size, best ranked model within the cluster for that screen, and best 0.8 ipTM + 0.2 pTM score and the mean 0.8 ipTM + 0.2 pTM score.

Cluster label	Cluster size	Best rank	Top ranked member ID	Weighted pTM score	Mean weighted pTM score
Cluster 1	2	1	ranked_1_Leptospira_borgpetersenii_LIC13259_no_his_and_Bos_taurus_C8_A8YXZ2	0.444952	0.342888
Cluster 2	7	5	ranked_5_Leptospira_wolffii_LIC13259_no_his_and_Bos_taurus_C8_A8YXZ2	0.291105	0.248936
Cluster 3	3	2	ranked_2_Leptospira_biflexa_LIC13259_no_his_and_Bos_taurus_C8_A8YXZ2	0.288889	0.275645
Cluster 4	3	6	ranked_6_Leptospira_interrogans_LIC13259_no_his_and_Bos_taurus_C8_A8YXZ2	0.292998	0.257545
Cluster 5	3	1	ranked_1_Leptospira_kmetyi_LIC13259_no_his_and_Bos_taurus_C8_A8YXZ2	0.278287	0.252847
Cluster 6	2	24	ranked_24_Leptospira_biflexa_LIC13259_no_his_and_Bos_taurus_C8_A8YXZ2	0.220437	0.220318
Cluster 7	3	9	ranked_9_Leptospira_biflexa_LIC13259_no_his_and_Bos_taurus_C8_A8YXZ2	0.249636	0.230437

Cluster 8	3	2	ranked_2_Leptospira_borgpetersenii_LIC13259_no_his_and_Bos_taurus_C8_A8YXZ2	0.346632	0.302082
Cluster 9	4	0	ranked_0_Leptospira_interrogans_LIC13259_no_his_and_Bos_taurus_C8_A8YXZ2	0.642092	0.501862
Cluster 10	2	3	ranked_3_Leptospira_wolffii_LIC13259_no_his_and_Bos_taurus_C8_A8YXZ2	0.314244	0.277615
Cluster 11	11	4	ranked_4_Leptospira_biflexa_LIC13259_no_his_and_Bos_taurus_C8_A8YXZ2	0.273	0.248145
Cluster 12	15	0	ranked_0_Leptospira_wolffii_LIC13259_no_his_and_Bos_taurus_C8_A8YXZ2	0.450413	0.276385
Cluster 13	19	0	ranked_0_Leptospira_borgpetersenii_LIC13259_no_his_and_Bos_taurus_C8_A8YXZ2	0.516721	0.259397
Cluster 14	18	0	ranked_0_Leptospira_kmetyi_LIC13259_no_his_and_Bos_taurus_C8_A8YXZ2	0.331109	0.250254
Cluster 15	14	4	ranked_4_Leptospira_interrogans_LIC13259_no_his_and_Bos_taurus_C8_A8YXZ2	0.336916	0.250455
